# Attenuation of osteoarthritis progression via locoregional delivery of Klotho-expressing plasmid DNA and Tanshinon IIA through a stem cell-homing hydrogel

**DOI:** 10.1186/s12951-024-02608-z

**Published:** 2024-06-10

**Authors:** Peng Wang, Zhibo Zhao, Ziyang Li, Xiao Li, Benzhao Huang, Xiaoqing Lu, Shimin Dai, Shishuo Li, Zhentao Man, Wei Li

**Affiliations:** 1 Department of Joint Surgery, Shandong Provincial Hospital, Shandong University, Jinan, Shandong 250021 P. R. China; 2 Department of Joint Surgery, Shandong Provincial Hospital Affiliated to Shandong First Medical University, Jinan, Shandong 250021 P. R. China; 3 Department of Orthopedic Surgery, Second Affiliated Hospital, Zhejiang University School of Medicine, Hangzhou, China; 4 College of Sports Medicine and Rehabilitation, Shandong First Medical University & Shandong Academy of Medical Sciences, Jinan, Shandong 250021 P. R. China; 5 Endocrine and Metabolic Diseases Hospital of Shandong First Medical University, Shandong Institute of Endocrine and Metabolic Diseases, Jinan, Shandong 250062 P. R. China

**Keywords:** Osteoarthritis, Senescent, Klotho, Stem cell-homing hydrogel, Cartilage rejuvenation

## Abstract

**Background:**

Osteoarthritis (OA) is an aging-related degenerative joint disorder marked by joint discomfort and rigidity. Senescent chondrocytes release pro-inflammatory cytokines and extracellular matrix-degrading proteins, creating an inflammatory microenvironment that hinders chondrogenesis and accelerates matrix degradation. Targeting of senescent chondrocytes may be a promising approach for the treatment of OA. Herein, we describe the engineering of an injectable peptide-hydrogel conjugating a stem cell–homing peptide PFSSTKT for carrying plasmid DNA-laden nanoparticles and Tanshinon IIA (pPNP + TIIA@PFS) that was designed to attenuate OA progression by improving the senescent microenvironment and fostering cartilage regeneration.

**Results:**

Specifically, pPNP + TIIA@PFS elevates the concentration of the anti-aging protein Klotho and blocks the transmission of senescence signals to adjacent healthy chondrocytes, significantly mitigating chondrocyte senescence and enhancing cartilage integrity. Additionally, pPNP + TIIA@PFS recruit bone mesenchymal stem cells and directs their subsequent differentiation into chondrocytes, achieving satisfactory chondrogenesis. In surgically induced OA model rats, the application of pPNP + TIIA@PFS results in reduced osteophyte formation and attenuation of articular cartilage degeneration.

**Conclusions:**

Overall, this study introduces a novel approach for the alleviation of OA progression, offering a foundation for potential clinical translation in OA therapy.

**Supplementary Information:**

The online version contains supplementary material available at https://doi.org/10.1186/s12951-024-02608-z.

## Introduction

Osteoarthritis (OA) is the most common form of arthritis, characterized by joint space narrowing, osteophyte formation, and subchondral bone sclerosis. These changes severely impact the quality of life for patients and create a significant burden for individuals, families, and society [1, 2]. The current standard of care for OA includes physical therapy to alleviate symptoms, medications such as nonsteroidal anti-inflammatory drugs and painkillers, and total joint replacement, which carries risks including postoperative infection [3]. However, there are no clinically available disease-modifying drugs that can prevent, slow down, or reverse the development of OA [4].

Research increasingly points to imbalanced catabolic and anabolic pathways and inadequate nutrient recycling mechanisms under stress conditions (e.g. aging) as the main causes of OA [5, 6]. Cellular senescence, which refers to the permanent termination of the cell cycle, is a common molecular mechanism that contributes to age-related OA [7, 8]. Adult cartilage has limited repair capacity and is prone to irreversible injury-related damage [9–11]. Therefore, the preservation of robust chondrocytes in cartilage is crucial for maintaining joint health. However, due to aging and mechanical stress, the ability of chondrocytes to maintain cartilage integrity and survive is gradually lost [12, 13]. Furthermore, senescent cells develop a pro-inflammatory phenotype called the senescence-associated secretory phenotype (SASP), which induces structural and functional changes in surrounding cells and tissues. These detrimental changes to the extracellular matrix further diminish the mechanical integrity and lubrication of cartilage, accelerating its wear and destruction [14]. Another mechanism of chondrocyte senescence in OA may involve intercellular communication, which negatively impacts neighboring healthy chondrocytes [15]. Recent studies have shown that senescent chondrocytes secrete elevated numbers of extracellular vesicles compared with normal cells [16, 17]. These vesicles function as signaling mediators that instigate senescence in proximal healthy cells [18, 19]. Studies also have documented the upregulated expression of two proteins that facilitate intercellular communication—cell communication network factor 1 (CCN1) and connexin 43—on the surface membranes of senescent chondrocytes [18, 20, 21]. Therefore, treatment strategies that improve the local senescent microenvironment and target the root causes of aging may hold promise for patients with OA.

Klotho, initially identified as an anti-aging molecule in mice, is downregulated in aging and OA, both in cartilage and synovium. Klotho reduces the expression of catabolic markers ZIP8 and MMP13 to maintain chondrocyte homeostasis [22], and also plays a crucial role in inhibiting extracellular matrix degradation [23–26]. Pre-clinical mouse models genetically engineered to express decreased Klotho protein levels display an aged phenotype, with accelerated development of OA [27]. The combined application of Klotho and soluble transforming growth factor-beta receptor 2 to maintain the chondrocytic phenotype has shown significant potential in rats [28]. Tanshinone IIA (TIIA), a purified component from *Salvia miltiorrhiza*, inhibits endogenous CCN1 secretion and CCN1-induced chondrocyte cluster formation and senescence alongside OA development [29, 30]. Such inhibition reverses the senescent phenotype of chondrocytes and fosters both de-differentiation and re-differentiation. The combination of pPNP and TIIA in our study is based on their complementary and synergistic effects. While both agents possess antioxidant and anti-aging properties, their mechanisms of action differ, providing a broader spectrum of protection against oxidative stress and cellular aging. pPNP, as a nanoparticle-based antioxidant, primarily offers intracellular ROS scavenging, whereas TIIA scavenges ROS both intra- and extracellularly and modulates inflammation and apoptosis pathways [31]. In light of the causal relationship between aging and OA pathogenesis, we postulated that the targeted delivery of the Klotho gene (*KL*) and TIIA to chondrocytes would effectively mitigate pain and facilitate the repair of damaged cartilage.

In addition to inhibiting cellular senescence, comprehensive joint rejuvenation and subsequent cartilage repair are essential for mitigating ongoing cartilage degradation in OA. Injectable hydrogels with favorable lubricating properties are highly preferred for joint protection and pain relief, while also facilitating the repair of cartilage defects [32]. These processes may be actualized through the recruitment of stem cells that differentiate into revitalized chondrocytes. In pursuit of this aim, we designed a self-assembling peptide (Ac-[KLDL]_3_-NH_2_) functionalized with the bone marrow homing peptide (BMHP) motif PFSSTKT [33]. This peptide self-assembles and forms a hydrogel with excellent mechanical properties and good biocompatibility in a physiological environment [34]. The porous structure of the hydrogel mimics the natural extracellular matrix, making it a promising medium for drug release and cellular delivery [35]. It can regulate mesenchymal stem cell (MSC) homing, thereby maintaining the integrity of articular cartilage.

**Fig. 1:**
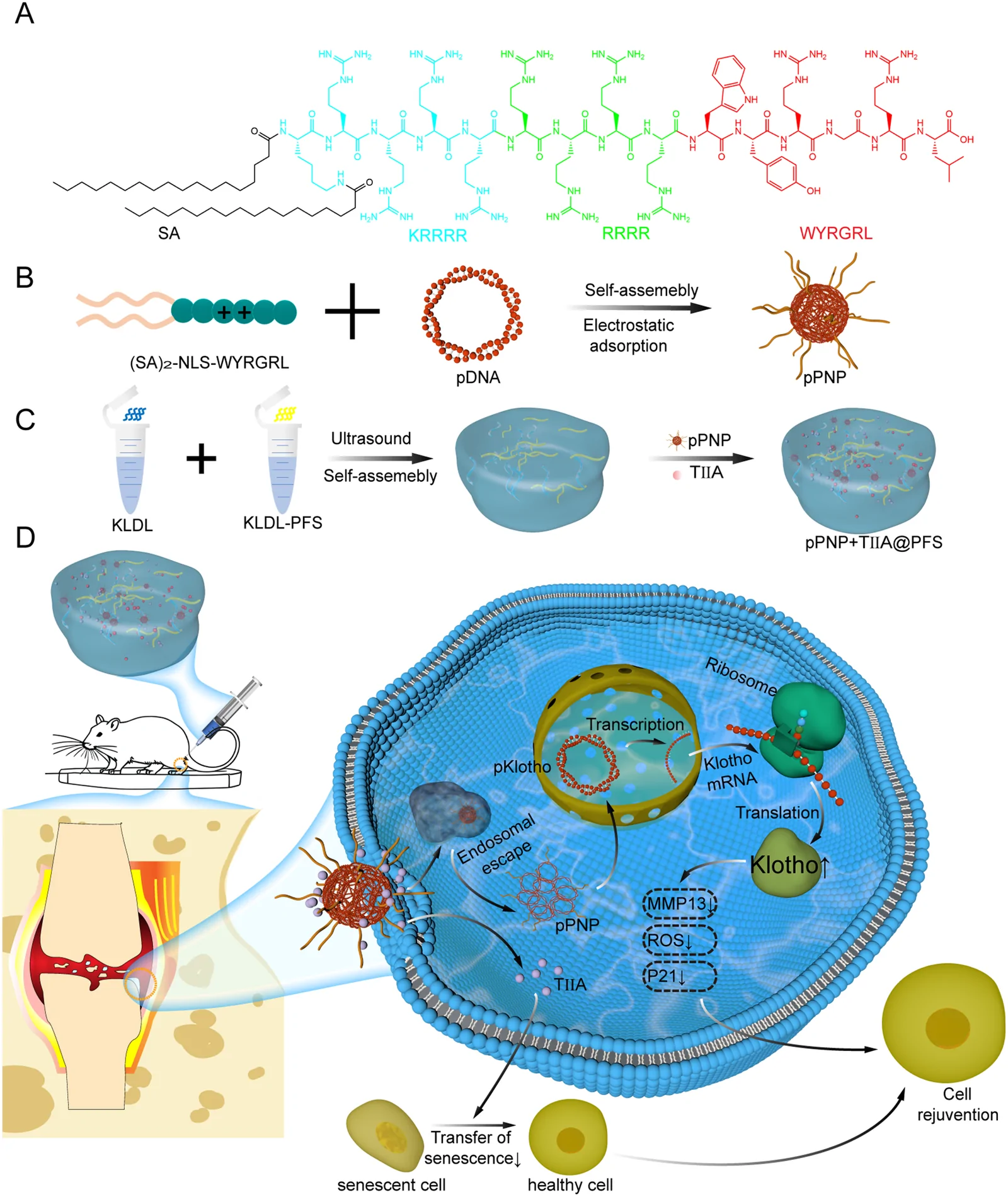
Schematic illustration of locoregional generation of Klotho protein to mitigate OA. (**A**) Chemical structure of the NPs featuring a nuclear localization sequence (NLS) peptide as the hydrophilic moiety and stearic acid (SA) as the hydrophobic domain. (**B**) Schematic illustration of the preparation of plasmid DNA (pDNA)-laden peptide NPs (pPNPs). (**C**) Schematic illustration of the pPNP coating on hydrogel. (**D**) The pPNP-gel generates Klotho protein to ameliorate the aging microenvironment and delay arthritis progression. In the anterior cruciate ligament transection (ACLT) rat model, this approach ameliorates arthritis and promotes osteogenesis

The non-specific distribution and non-targeted effects of gene therapy in vitro and in vivo hinder its clinical utility [21, 36]. Furthermore, the requirement to reduce the frequency of intra-articular injections, coupled with the rapid clearance of drugs directly administered into affected joints, restricts both the duration and efficacy of treatment [37, 38]. To address these challenges, we engineered a nanoparticle (NP) hydrogel superstructure designed for the localized, in situ overexpression of Klotho protein within the articular cavity (Fig. 1). This strategy was designed to enable specific targeting of chondrocytes and to promote cartilage regeneration by recruiting stem cells from bone marrow and synovium, mitigating further deterioration [39]. Our findings suggest that this nanohydrogel superstructure ameliorates the senescent microenvironment, thereby slowing the progression of OA and facilitating the recruitment of stem cells for cartilage repair. These results offer a potential therapeutic approach for the treatment of OA and warrant further evaluation through clinical trials.

## Materials and methods

### Cell isolation and culture

Cartilage samples extracted from rat femurs for the isolation of rat chondrocytes were subsequently cultured in Dulbecco’s Modified Eagle’s Medium (DMEM)/Nutrient Mixture F-12 supplemented with 10% fetal bovine serum (FBS). BMSCs were procured from the Institute of Biochemistry and Cell Biology, Shanghai Institutes for Biological Sciences, Chinese Academy of Sciences. All cells were maintained in a humidified incubator with a 5% CO_2_ atmosphere at 37 °C.

### Synthesis and characterization of pPNP

(SA)_2_-KRRRR-NLS-WYRGRL was custom-synthesized by Shanghai Apeptide Co. Ltd. The NPs (2 mg) were dissolved in 10 µL dimethyl sulfoxide and diluted with 1 mL diethylpyrocarbonate-treated water solution containing 35 µL pDNA (weight ratio of peptide-SA monomer: pDNA = 10:1). The mixture was vortexed to obtain pPNP. The dynamic light scattering and zeta potential analysis of the resultant nanomicelles were conducted with a Zeta Sizer Nano ZS90 (Malvern Instruments Ltd., UK). The morphology of the pPNP was characterized using TEM (JEM-1200 EX II electron microscope) after negative staining with 2% phosphotungstic acid.

### Preparation and characterization of stem cell-homing hydrogels

Self-assembling peptide KLDL-12, comprising the sequence Ac-(KLDL)_3_-NH_2_, and the newly designed functionalized peptide KLDL-PFS, comprising the sequence Ac-(KLDL)_3_-GG-PFSSTKT-NH_2_, were custom synthesized by QY Biochem Ltd. (Shanghai, China). The two peptide powders were dissolved in 10% (w/v) sterile sucrose solution at a peptide concentration of 1% (10 mg/mL), then sonicated for 30 min at a frequency of 40 kHz in a DSA50-GL1 ultrasonic cleaner (DESEN, Shenzhen, China). Equal volumes of the KLDL-12 and KLDL-PFS solutions were mixed to maintain a 1% concentration. The individual and mixed-peptide solutions were then allowed to stand for 30 min to achieve complete gelation. The gels were characterized by SEM (FEI Quanta FEG250, Hillsboro, USA) using field emission scanning (accelerating voltage, 8 kV; working distance, 7.2 mm), TEM, FTIR, and circular dichroism. Hydrogel rheology was measured on a rheometer (Anton-Paar MCR302, Graz, Austria).

### Agarose gel electrophoresis

Agarose gel electrophoresis was used to study the complete complexation of (SA)_2_-peptide with pKlotho. NPs were formulated using (SA)_2_-peptide and pDNA in the following ratios: 2:1, 5:1, 10:1, 15:1, and 20:1. A 10-µL aliquot containing 0.3 µg of pDNA was combined with DNA loading buffer and introduced into parallel wells of a 1.2% (w/v) agarose gel. The gel also included 0.5 µL/mL NAGreen dye (Beyotime, Shanghai, China) in Tris-acetate-EDTA buffer. Electrophoresis was carried out at 100 V for 60 min, and gel retardation patterns were captured using an Amersham Imager 600 RGB (Amersham plc, UK).

### Cellular uptake and NP-mediated gene transfection in vitro

Chondrocytes were exposed to each NP formulation to evaluate the cellular uptake of pPNP both qualitatively and quantitatively. For the in vitro gene transfection assays, chondrocytes, BMSCs, or RAW264.7 cells were treated with either saline or pPNP. Following 48 h of incubation, the proportion of EGFP-positive cells was determined via flow cytometry. For confocal laser scanning microscopy observations, cell nuclei were stained with DAPI prior to analysis with a CLSM 780 (Carl Zeiss Inc., Oberkochen, Germany).

### Transcriptome sequencing and data analysis

Following pPNP-mediated treatment, chondrocytes were processed with TRIzol reagent (Beyotime) and preserved at − 80 °C until sequencing. RNA sequencing was conducted on an Illumina HiSeq X10 system (Illumina, San Diego, CA, USA). Gene expression levels were calculated as the log_10_-transformed value of [transcripts per million reads (TPM) + 1]. The RNA sequencing data were normalized via the fragments per kilobase per million reads method.

### Detection of inflammation and senescence biomarkers in chondrocytes

Standard immunofluorescence staining was performed using primary antibodies against P16^INK4a^, MMP13, and COL2A1. The proportions of cells positive for P16^INK4a^, MMP13, and COL2A1 were counted by ImageJ software. Western blot was conducted to verify the expression of proteins under different interventions, including control, pPNP, T II A, and pPNP + T II A (see antibody details in Table S2). Relative gene expression was quantified using the 2^−ΔΔCt^ method with β-actin as the reference gene. The primer sequences employed for these experiments are provided in Table S3.

### SA–β-galactosidase staining

SA–β-galactosidase staining was performed using a SA–β-gal staining kit (Beyotime) in accordance with the manufacturer’s instructions. Chondrocytes in three random microscope images were counted using ImageJ software.

### ROS scavenging

Chondrocytes were seeded onto 12-well plates and stimulated with lipopolysaccharide (500 ng/mL). Following treatment with different NP formulations for an additional 24 h, the treated chondrocytes were incubated with 2’,7’- dichlorodihydrofluorescein diacetate (DCFH-DA; 10 µM) and then observed under fluorescent microscopy to detect intracellular ROS.

### Transwell migration assay

BMSCs were seeded onto the upper chamber of a Transwell system and cultured in serum-free, low-glucose DMEM. The lower chamber was supplemented with DMEM containing 10% FBS. After a 24-hour incubation, MSCs on the upper membrane surface were removed, and those that had migrated to the lower layer were fixed with 4% paraformaldehyde and stained with 1% crystal violet.

### In vitro assays of pPNP + TIIA@PFS on chondrogenesis

BMSCs were seeded in 12-well plates and chondrogenically induced as described above for 7 days. GAG formation was detected using Alcian blue staining. Indicators of chondrogenesis were assessed through WB (Aggrecan, SOX9, COL2A1), qRT-PCR (Aggrecan, SOX9, COL2A1), and immunofluorescence (Aggrecan, COL2A1) analyses.

### Evaluation of pPNP + TIIA@PFS biocompatibility in vitro

BMSCs and chondrocytes were seeded at a density of 5 × 10^4^ cells/mL onto hydrogels in 24-well culture plates. After blocking, actin filaments and nuclei were stained with phalloidin and DAPI, respectively. The stained samples were imaged with a Zeiss LSM880 confocal microscope equipped with a Plan-Apochromat 20×/0.80 M27 objective lens. Cell proliferation was assessed using a Cell Counting Kit 8 (CCK-8) assay, and results were normalized to the optical density (OD)_450 nm_ values of the blank hydrogel on day 1 post-seeding, expressed as relative proliferation rates.

### Evaluation of pPNP + TIIA@PFS biocompatibility, degradability, persistence, and biodistribution in vivo

Blood samples (200 µL) were collected from the eye socket venous plexus at 1, 2, and 4weeks post-injection for hematological analysis. Subsequently, the animals were euthanized using CO_2_ inhalation, and major organs were harvested and fixed for 24 h for histology.

To assess degradability, Cy5-labeled pPNP@PFS were injected into the distal femur. Anesthesia was induced using inhaled isoflurane (2%), and in vivo fluorescence imaging was conducted using the IVIS Lumina II system (PerkinElmer, Hopkinton, MA, USA).

Evaluation of anti-inflammatory and anti-aging effects of pPNP + TIIA@PFS in vivo.

Bone tissues were imaged using a SkyScan 1172 micro-CT system at a resolution of 36 μm and a rotation step of 0.15°/180°. Images were reconstructed using SkyScan NRecon software and further analyzed with CTAN software to generate 3D representations. Metrics were computed using CT-Analyser software (version 1.11; SkyScan). Markers of inflammation (MMP13) and senescence (P21) were evaluated using immunohistochemical staining at 7 and 10weeks post-surgery.

### Evaluation of chondrogenesis in vivo

Bone tissues were collected at 7 and 10weeks post-hydrogel injection, fixed in 4% paraformaldehyde, decalcified using EDTA, dehydrated through graded ethanol solutions, and embedded in paraffin. Paraffin-embedded tissue sections were stained with H&E and safranin O/Fast Green to evaluate new cartilage formation. Sections were also incubated overnight with primary antibodies against CD90 (Abcam, Cambridge, UK) at 4 °C, followed by a 1-hour incubation with Alexa Fluor-labeled secondary antibodies (Life Technologies, Waltham, MA) at room temperature. DAPI was used for counter-staining.

### Statistical analysis

Data are presented as means, with error bars indicating the standard deviation (SD) from independent samples. Animal groupings were randomized prior to treatment initiation. Statistical comparisons were made using one-way analysis of variance, two-tailed Student’s t-test, or Fisher’s exact test, as specified in the figure legends. All statistical analyses were performed using Prism software (GraphPad). A P value < 0.05 was considered to indicate a statistically significant difference.

## Results

### Synthesis and characterization of the plasmid DNA (pDNA)-laden peptide NP (pPNP) + TIIA@PFS superstructure

**Fig. 2:**
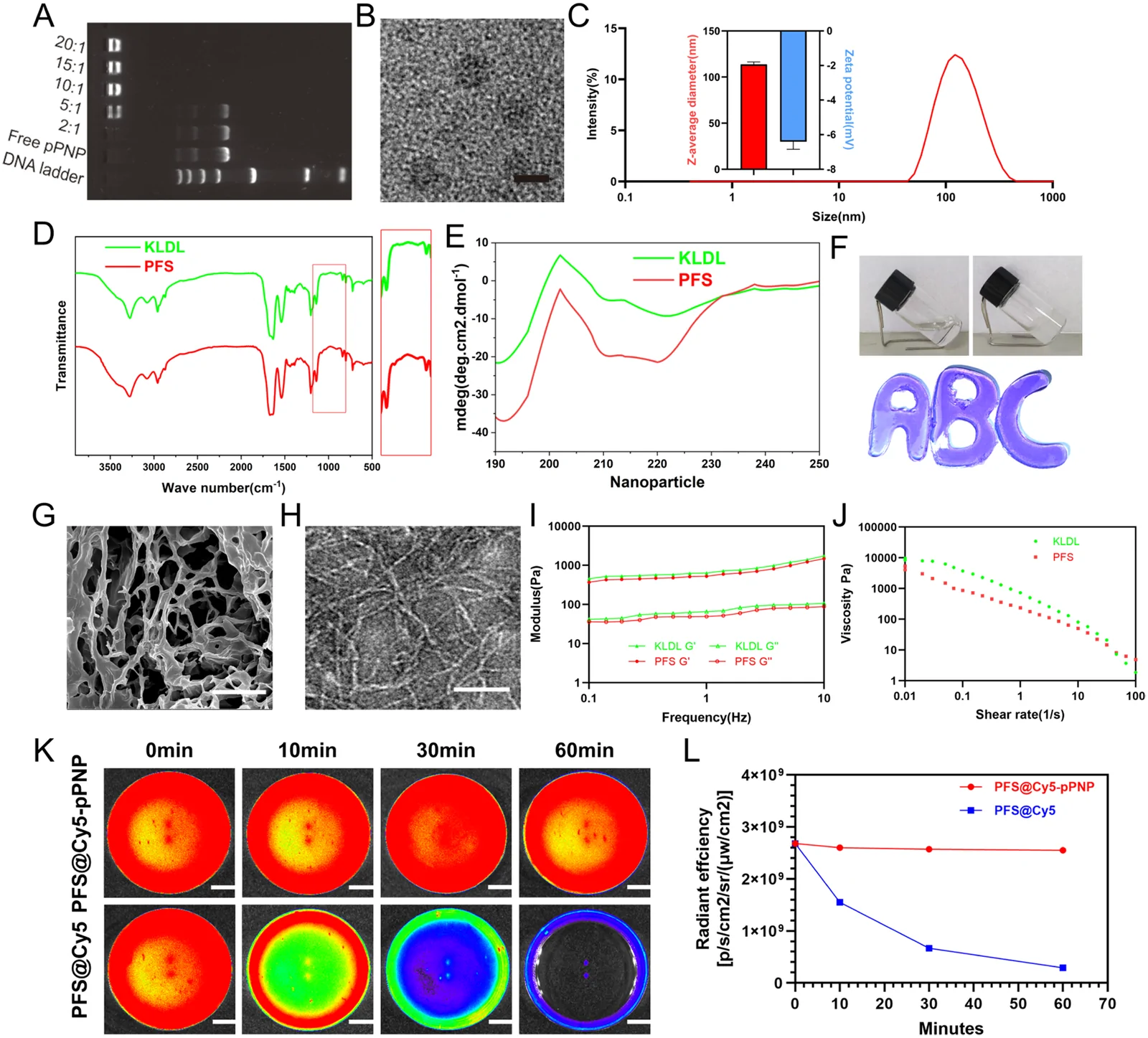
Design and characterization of the pPNP coating. (**A**) Gel retardation assay of pPNPs. (**B**) TEM images of pPNPs with a weight ratio of SA-peptide: pDNA of 10:1. Scale bar, 100 nm. (**C**) Zeta potential and size distributions of pPNPs with a weight ratio of SA monomers: pDNA of 10:1. (**D**, **E**) FTIR spectra (**D**) and circular dichroism spectra (E) of the hydrogels. (**F**) Photos of peptide solutions and the hydrogels. (**G**, **H**) SEM (**G**; scale bar, 20 μm) and TEM (**H**; scale bar, 500 nm) images of the hydrogels. (**I**) Changes in shear viscosity with the increased shear rate of the hydrogels. (**J**) Rheology traces of the hydrogels. (**K**) Representative fluorescence images of hydrogels with PFS@Cy5 and PFS@Cy5-pPNP collected via a live imaging system at 0, 10, 30, and 60 min. Scale bar, 600 μm. (**L**) Quantitative analysis of a time course of fluorescence radiant efficiency of PFS@Cy5 and PFS@pPNP hydrogels (*n* = 3). Data are presented as the mean ± SD

We confirmed the chemical structure of the pPNP through high-performance liquid chromatography (HPLC) and electrospray ionization mass spectrometry (ESI-MS) (Fig. S1). Figure 2A illustrates that complete pDNA loading by NPs was achieved when the weight ratio of the peptide-stearic acid (SA) monomer to pDNA exceeded 10:1. Transmission electron microscopy (TEM) images revealed a well-defined, spherical structure for the pPNP, with an average diameter of 110.5 nm (Fig. 2B, C). Gel retardation assays were performed to evaluate NP stability and confirm that the pPNP remained stable under different conditions (Fig. s2).

To extend the retention time of the plasmid in the knee joint, we engineered a drug delivery system based on a self-assembling peptide hydrogel. Particularly, a cationic functional peptide PFSSTKT was introduced into the hydrogel for more sustained and stable release and for endogenous stem cell recruitment. Fourier-transform infrared spectroscopy (FTIR) (Fig. 2D) and Raman spectroscopy (Fig. S3) were employed to analyze the secondary structural transitions of the KLDL hydrogel and KLDL hydrogel conjugating PFSSTKT peptide. Characteristic peaks at 940 cm^− 1^ in the FTIR for both KLDL and PFS corroborated the vibrational behavior of chemical bonds within the hydrogel. Furthermore, component blending had no adverse effect on the β-sheet structure or nanofiber formation (Fig. 2E). Chemical structures and mass spectra for the self-assembling peptide hydrogel are presented in Fig. S4. As shown in Fig. 2F, the hydrogel exhibited transparency and self-supporting properties. Scanning electron microscopy (SEM) and TEM analyses revealed that the nanofibers displayed a randomly entangled porous structure (Fig. 2G, H). Dynamic sweep rheological assays were performed to evaluate the rheological attributes of the tested hydrogels, all of which featured an energy storage modulus (G’) that surpassed the loss modulus (G’’) across the examined frequency range (Fig. 2I). An elevated energy storage efficiency was indicative of successful hydrogel formation. Upon varying the shear rate from 0.01 to 100 s^− 1^, a marked reduction in viscosity was noted, confirming that the shear-thinning hydrogels were amenable to injection (Fig. 2J). These data substantiated the injectability and self-healing capabilities of the hydrogels. As shown in Fig. 2K and L, Cy5-labeled pPNP exhibited a uniform distribution and retention within the PFS-based hydrogel. Lastly, the in vitro biodegradability of the peptide hydrogels was assessed, with each exhibiting an almost linear degradation pattern when subjected to proteinase K (Fig. S5A). Additionally, NP-encapsulated pDNA exhibited substantially prolonged release compared to free pDNA over the same duration (Fig. s5B).

**pPNP-mediated*KL*programming in chondrocytes**.

**Fig. 3:**
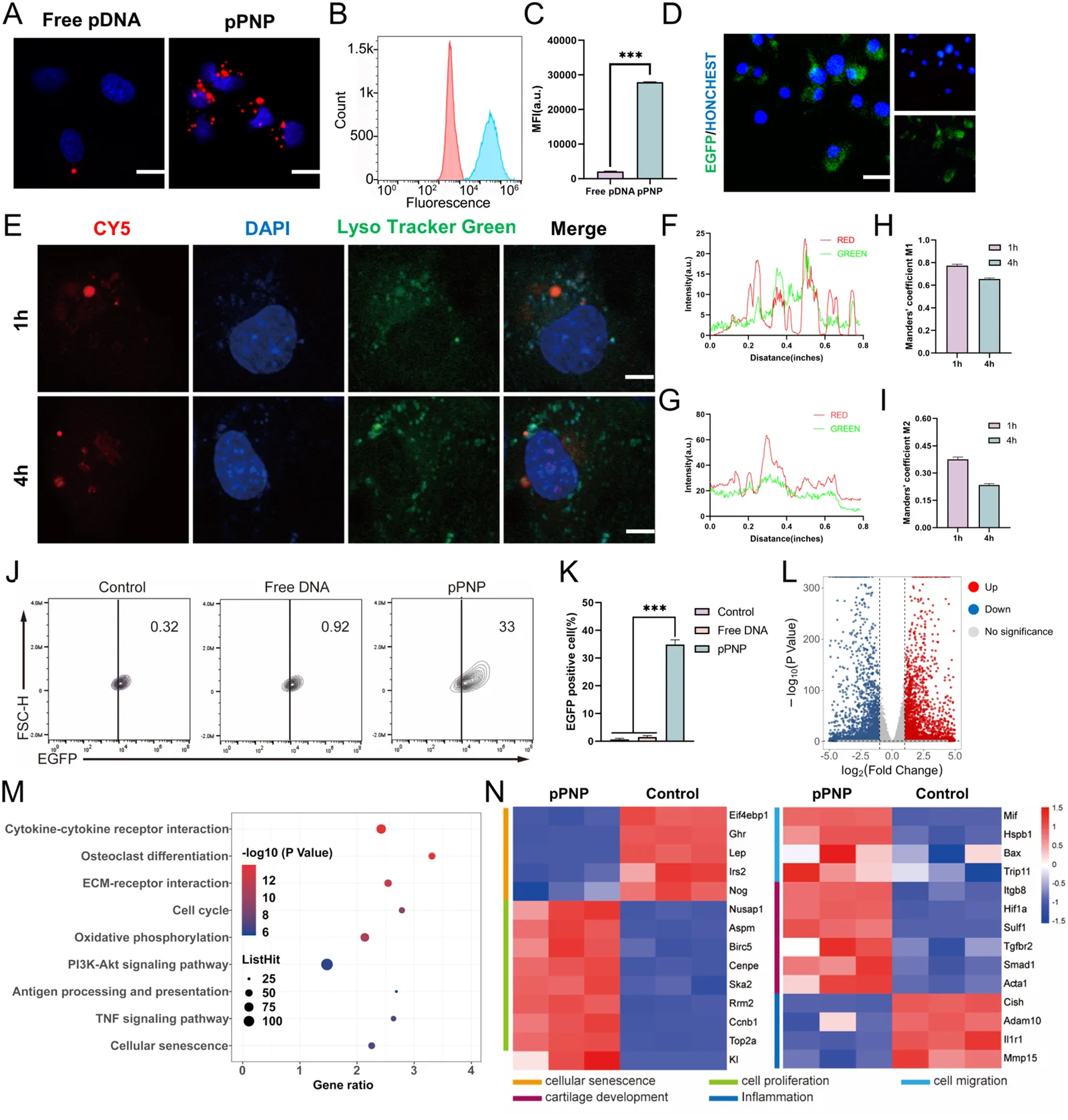
pPNP-mediated *KL* programming in chondrocytes. (**A**) Confocal images of chondrocytes treated with free pDNA or pPNP. Nuclei were counterstained with DAPI (blue). Scale bar, 20 μm. (**B**, **C**) Cellular uptake of free pDNA or pPNP by chondrocytes, as measured through flow cytometry analysis (*n* = 3). (**D**) Representative microscopy image of EGFP-positive chondrocytes. Scale bars, 25 μm. (**E**) Typical confocal images of chondrocytes incubated with pPNPs for 1–4 h at 37 °C. Nuclei nuclei were stained with DAPI (blue), endosomes/lysosomes were stained with LysoTracker Green (green), and pDNAs were labeled with Cy5 (red). Scale bars, 10 μm. (**F**–**I**) Quantitative analysis of the co-localization of Cy5-labeled pDNA with LysoTracker Green-labeled endosomes/lysosomes (*n* = 3). (**J**–**K**) Percentage of EGFP-positive chondrocytes treated with free pDNA or pPNP (*n* = 3). (**L**) Volcano plot of genes differentially expressed in untreated versus pPNP-treated chondrocytes. (**M**) KEGG pathway analysis of the differentially expressed genes. (**N**) Heatmap of differentially expressed senescence- and inflammatory-related genes between untreated and pPNP-transduced chondrocytes (*n* = 3). Data are presented as the mean ± SD. ***P < 0.001

The in vitro transfection of chondrocytes with plasmids encapsulated in NPs substantially augmented cellular gene internalization (Fig. 3A). Flow cytometry and confocal microscopy further corroborated that the efficient delivery of genes into chondrocytes by cartilage-targeting pPNP (Fig. 3B–D). Consistently, immunofluorescence staining of the cartilage tissues around a pPNP-hydrogel complex depicted well-dispersed pPNP (Fig. S6). The expansive distribution of Cy5-pPNP in chondrocyte cytoplasm, which increased with incubation time, signaled successful lysosomal escape and cytoplasmic entry of NPs (Fig. 3E). To substantiate these observations, quantitative co-localization analysis of pPNPs with endonucleases and lysosomes in confocal fluorescence images was performed using Manders’ coefficients M1 and M2 (Fig. 3F–I).

Subsequently, the specificity of the Col2 promoter in regulating *KL* expression in chondrocytes was examined. Flow cytometry revealed significantly increased the number of enhanced green fluorescent protein (EGFP)-positive chondrocytes following incubation with pPNP compared to incubation with free pDNA, as shown in Fig. 3J and S7. These results confirmed the specificity of the Col2 promoter in driving EGFP expression specifically in chondrocytes, thus mitigating off-target effects. To explore the characteristics of Klotho-overexpressing chondrocytes, we conducted transcriptomic analysis after pPNP-mediated treatment. Volcano plots of differentially expressed genes in chondrocytes with and without treatment with pPNP showed a distinct segregation in gene expression (Fig. 3L). Kyoto Gene and Genome Encyclopedia (KEGG) pathway analysis revealed activation of genes involved in major signaling pathways, including cellular senescence, the tumor necrosis factor (TNF) signaling pathway, and cytokines (Fig. 3M). Changes in the expression of rejuvenation-related and inflammatory factors between untreated and pPNP-treated chondrocytes were further analyzed and visualized (Fig. 3N). Heatmap analysis of secreted factors revealed upregulation of several rejuvenation-related and anti-inflammatory genes, including *Mif*, *Ska2*, *Bax*, *Aspm*, *Hif1a*, and *Smad1*, in the pPNP group. Overall, pPNP treatment appears to be beneficial for revitalizing Klotho protein activity and enhancing the expression of anti-inflammatory, senescence-delaying, and matrix-generating properties in chondrocytes.

### Alleviation of chondrocyte senescence and attenuation of OA progression via Klotho protein in vitro

**Fig. 4:**
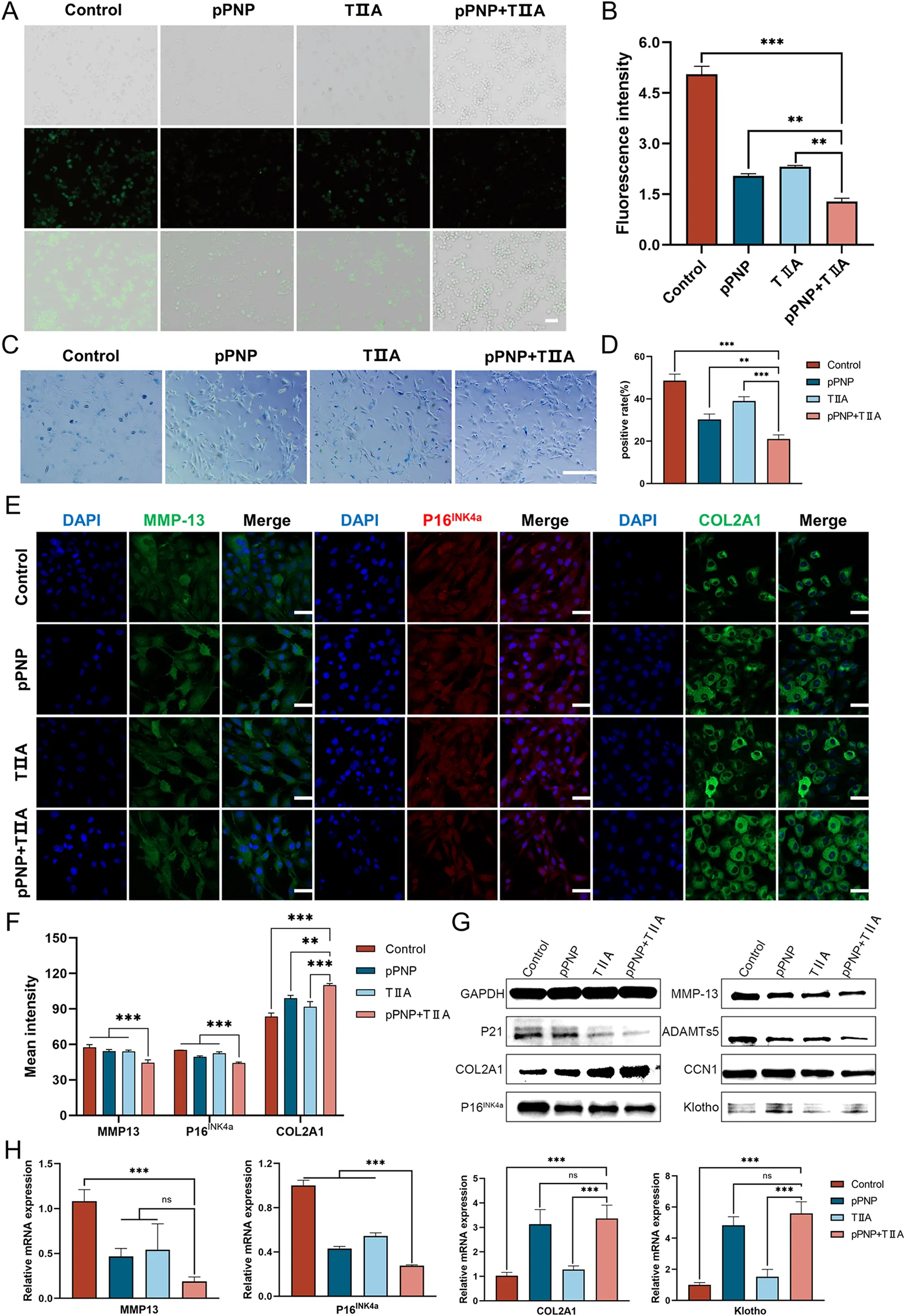
Klotho-mediated alleviation of senescence and rescue of OA cartilage degeneration in rat chondrocytes. (**A**) DCFH-DA fluorescence staining to detect chondrocytes with oxidative stress damage and measure ROS scavenging. Scale bar, 100 μm. (**B**) Quantitative analysis of ROS clearance (*n* = 3). (**C**) SA-β-galactosidase staining images of cultured chondrocytes in the Control, pPNP, T II A, and pPNP + T II A treatment groups. Scale bar, 500 μm. (**D**) Quantification of SA-β-galactosidase positivity (*n* = 3). (**E**) Immunofluorescence staining of Col2A1, MMP13, and P16^INK4a^ in rat chondrocytes. Scale bar, 40 μm. (**F**) Quantitative analysis of fluorescence intensity (*n* = 3). (**G**) Expression of Col2A1, MMP13, CCN1, ADAMTS5, Klotho, P16^INK4a^, and P21 at 2 days post-transfection of rat chondrocytes with different cultures, as determined by WB (*n* = 3). (**H**) qPCR evaluation of the expression of Col2A1, MMP13, Klotho, and P16^INK4a^ at 2 days post-transfection of rat chondrocytes with different cultures (*n* = 3). Data are presented as the mean ± SD. **P < 0.01, ***P < 0.001. NS, not significant

We assessed four chondrocyte treatment groups: control, pPNP, TIIA, and pPNP + TIIA. The pPNP + TIIA treatment group showed the highest reactive oxygen species (ROS) scavenging activity (Fig. 4A, B). We further investigated whether Klotho protein overexpression could inhibit senescence in these groups. The presence of Klotho protein in the pPNP and pPNP + TIIA groups significantly reduced the numbers of SA-β-galactosidase-positive cells (Fig. 4C, D). Immunofluorescence results in chondrocytes demonstrated that Klotho protein (Fig. S8) enhanced the expression of Col2A1 while inhibiting the expression of MMP13 and P21 (Fig. 4E, F).

We investigated the etiological roles of multiple factors involved in OA, including extracellular matrix components of cartilage (Col2A1), catabolic enzymes (MMP13, Adamts5), and markers of cellular senescence (P16^INK4a^ and P21) in vitro. Protein immunoblotting (WB) and quantitative real-time polymerase chain reaction (qRT-PCR) were employed to assess these molecular mechanisms. Our data revealed that Klotho overexpression led to an upregulation of Col2A1 and a concurrent downregulation of Adamts5, MMP13, P16^INK4a^, and P21 (Fig. 4G–H, S9) in rat chondrocytes. These observations suggested that Klotho acts in a protective manner by diminishing the levels of catabolic enzymes and senescence-associated genes while enhancing the synthesis of cartilage extracellular matrix, thereby ameliorating OA symptoms. CCN1 protein levels were notably reduced upon treatment with TIIA, confirming the potential inhibitory effects of TIIA on CCN1 expression in senescent cells. Collectively, these findings highlight the therapeutic potential of Klotho protein in mitigating OA-related pathology.

### pPNP + TIIA@PFS-induced recruitment and chondrogenic differentiation of bone marrow-derived mesenchymal stem cells (BMSCs)

**Fig. 5:**
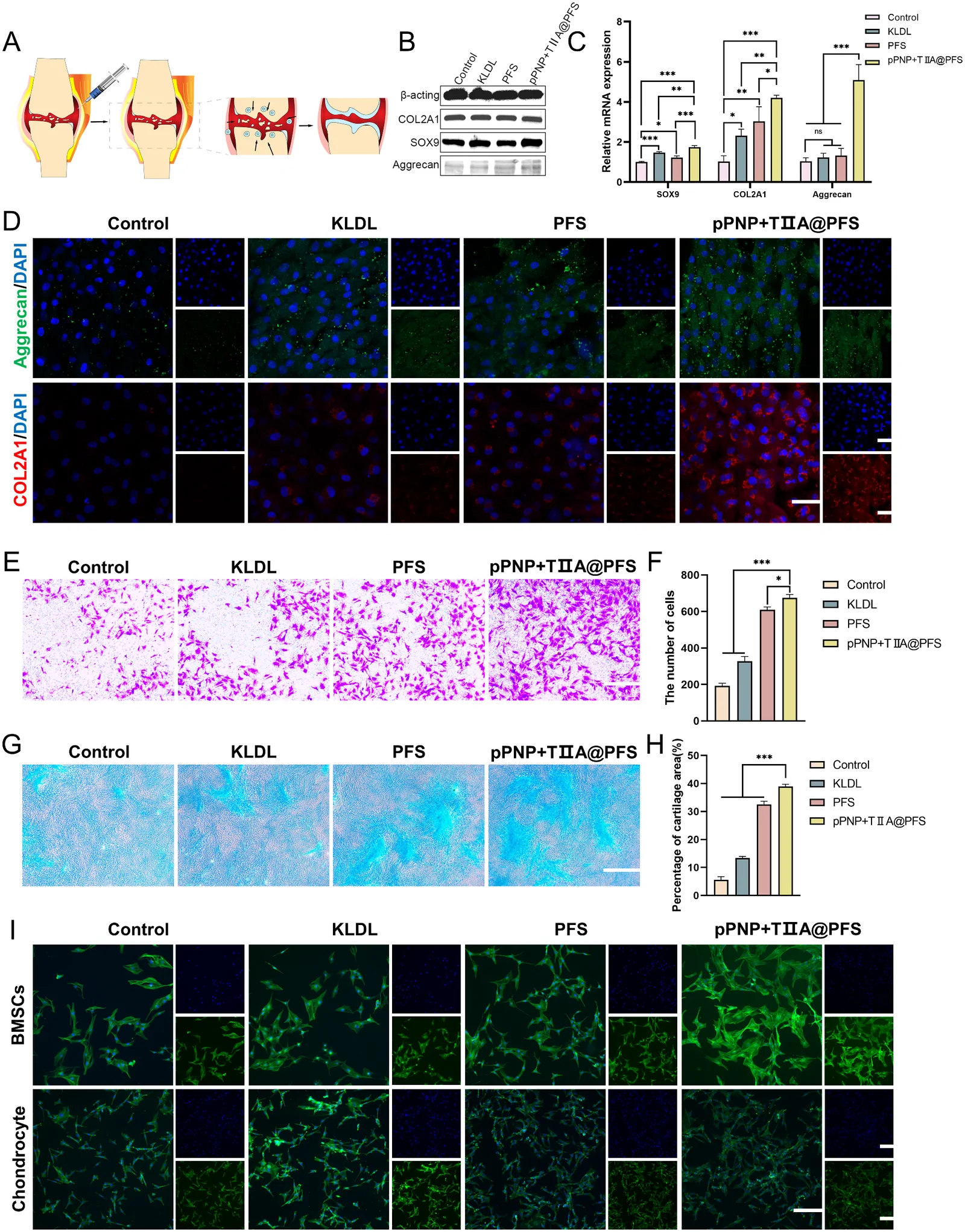
PFS@pPNP-mediated induction of BMSC recruitment and promotion of chondrogenic differentiation. (**A**) Schematic illustration of the recruitment process pattern of BMSCs during cartilage repair. (B, C) WB and qRT-PCR analyses of protein (**B**) and mRNA (**C**) levels of Sox9, Aggrecan, and Col2A1 in BMSC pellets in the indicated treatment groups (*n* = 3). (**D**) Immunofluorescence staining of Aggrecan and Col2A1 in BMSCs cultured on KLDL or PFS for 7 days. Scale bar, 40 μm. (**E**) Representative images of the Transwell bottom membrane stained with crystal violet after 24-h culture in the indicated treatment groups. Scale bar, 500 μm. (**F**) Quantification of cells observed at the bottom of the membrane (*n* = 3). (**G**) Alcian blue staining in BMSC pellets after 7-day culture in the indicated treatment groups. Scale bar, 250 μm. (**H**) Quantification analysis of chondrocyte area following Alcian blue staining (*n* = 3). (**I**) Alexa Fluor 488 phalloidin and DAPI staining showing morphology of BMSCs and chondrocytes grown under KLDL or PFS. Scale bar, 200 μm. Data are presented as the mean ± SD. *P < 0.05, **P < 0.01, ***P < 0.001. NS, not significant

The PFS peptide demonstrated a positive impact on BMSC recruitment, thereby facilitating the repair of damaged cartilage in OA (Fig. 5A). To build upon this observation, we assessed the influence of pPNP + TIIA@PFS on the chondrogenic differentiation of BMSCs. The pPNP + TIIA@PFS-treated group displayed elevated levels of Sox9, Col2A1, and aggregated proteoglycans relative to the other groups (Fig. 5B, C). Immunofluorescence analyses further confirmed increased expression levels of Col2A1 and Aggrecan in cells treated with PFS hydrogel (Fig. 5D).

Furthermore, compared to cells treated with KLDL peptide-based hydrogel, PFS peptide-based hydrogel treatment resulted in significantly increased cell migration to the lower side of the porous Transwell membrane (Fig. 5E, F). Although all samples stained positively for Alcian blue (Fig. 5G, H), those treated with pPNP + TIIA@PFS demonstrated strongest synthesis of glycosaminoglycan (GAG). Additionally, pPNP + TIIA@PFS enhanced BMSC proliferation, underscoring its efficacy in maintaining stem cell numbers and vitality (Fig. S10). Collectively, these findings suggest that PFS@pPNP promotes chondrogenic differentiation of BMSCs. Phalloidin fluorescence staining revealed that extensive cell spreading is indicative of stable attachment and cellular health (Fig. 5I). Overall, these data substantiate the notion that pPNP + TIIA@PFS augments both the proliferation and migration of BMSCs and stimulates their differentiation into chondrocytes, thus contributing to the repair of cartilage defects induced by OA.

**pPNP + TIIA@PFS-mediated alleviation of senescence and mitigation of OA progression after anterior cruciate ligament transection (ACLT) surgery**.

**Fig. 6:**
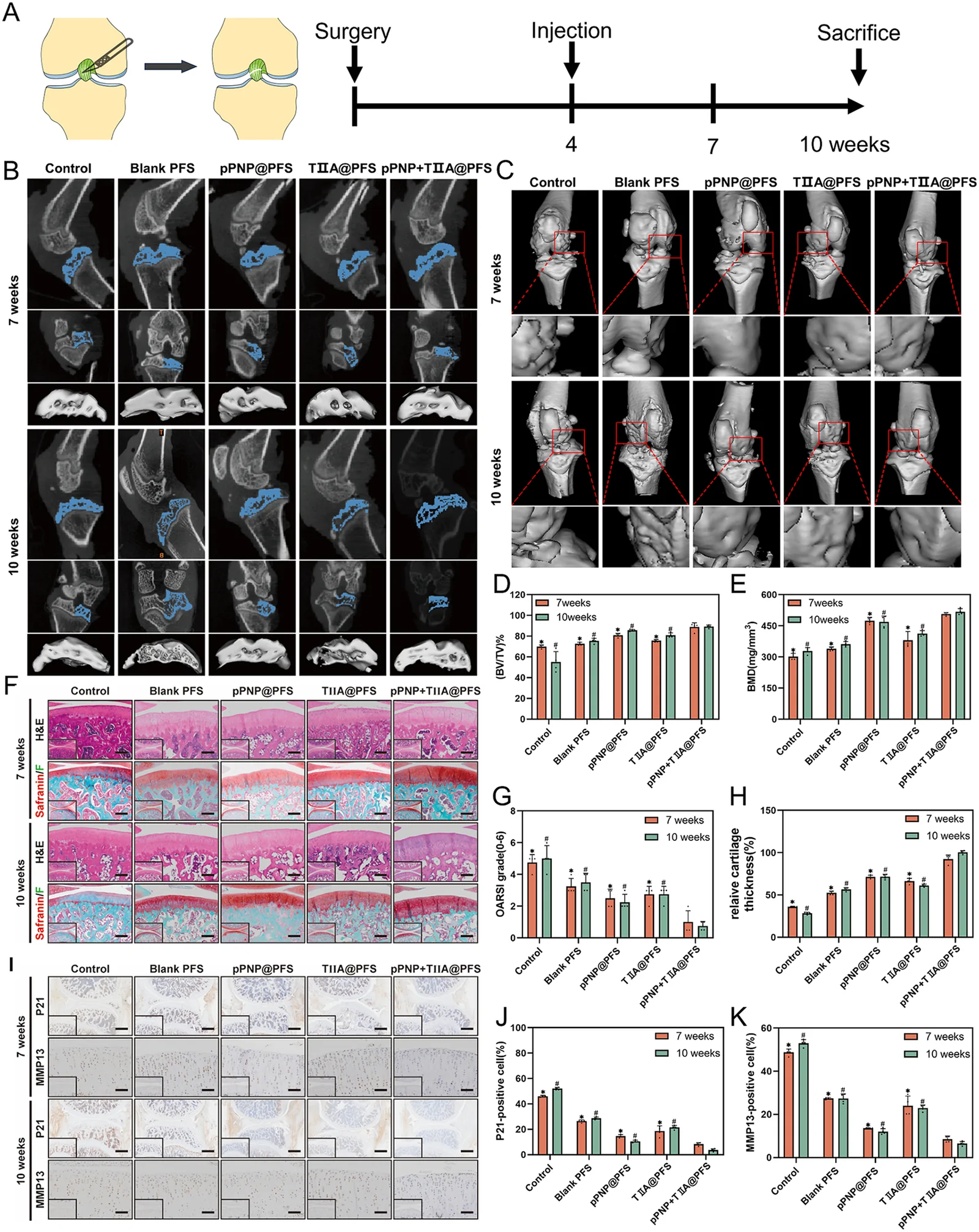
pPNP + T II A@PFS-mediated rescue of OA cartilage degeneration post-ACLT surgery in rats. (**A**) Schematic illustration of the experimental design. (**B**) Representative 2D images in the sagittal and coronal planes, and 3D images of subchondral bone reconstructed by micro-CT. (**C**) Three-dimensional images of rat knee joints showing the abnormal growth of osteophytes in the control, Blank PFS, pPNP@PFS, T II A@PFS, and pPNP + T II A@PFS groups at 7 and 10 weeks post-ACLT. (**D**, **E**) Quantitative statistics of the percentage of BV/TV (**D**) and total BMD (**E**) on micro-CT (*n* = 4). (**F**) Representative rat knee joint images stained with safranin O/Fast Green and H&E at 7 and 10 weeks. Scale bar, 200 μm. (**G**, **H**) OARSI scores of rat joints (**G**) and relative cartilage thickness (**H**) at 7 and 10 weeks (*n* = 4). (**I**) Representative immunohistochemistry staining images of P21 (Scale bar, 850 μm) and MMP13 (Scale bar, 200 μm) in rat knee joints from the control, Blank gel, pPNP@PFS, T II A@PFS, and pPNP + T II A@PFS groups at 7 and 10 weeks. (**J**, **K**) Quantification of cells with histological positivity for P21 (**J**) and MMP13 (**K**) (*n* = 4). Data are presented as the mean ± SD. *P < 0.05 versus pPNP + TIIA@PFS group at 7 weeks, ^#^*P* < 0.05 versus pPNP + TIIA@PFS group at 10 weeks, **P < 0.01, ***P < 0.001. NS, not significant

The pPNP + TIIA@PFS was injected into the rat knee joint at 4 weeks post-ACLT to assess its therapeutic efficacy against OA (Fig. 6A). Micro-computed tomography (CT) analysis of untreated control rats at 7 weeks post-ACLT revealed increased osteophyte formation, suggesting an aberrant bone-healing process. In contrast, the pPNP + TIIA@PFS combination treatment group displayed a marked reduction in osteophytes within the knee joint (Fig. 6B, C). As shown in Fig. 6D and E, the pPNP + TIIA@PFS group also manifested higher tissue bone mineral density (BMD) and a superior bone volume-to-tissue volume ratio (BV/TV). Furthermore, the knees of the pPNP + TIIA@PFS group showed minimal indications of cartilage degradation at weeks 7 and 10 post-ACLT, as evidenced by hematoxylin and eosin (H&E) and safranin O/fast green staining (Fig. 6F). Furthermore, there was a noteworthy reduction in Osteoarthritis Research Society International (OARSI) scores [40] in the pPNP + TIIA@PFS combination treatment group compared to the Control group (Fig. 6G, H).

To further explore the balance between senescence and catabolic activity in cartilage, immunohistochemical staining techniques were employed. In the pPNP + TIIA@PFS combination treatment group at 10 weeks post-ACLT, the levels of MMP13 and P21 were significantly reduced compared to untreated control rats (Fig. 6I). Between the 7-week and 10-week checkpoints, additional declines in P21-positive and MMP13-positive cells were observed within the pPNP + TIIA@PFS group (Fig. 6J, K). These results indicated that pPNP + TIIA@PFS may regulate the senescent microenvironment around damaged cartilage, promoting its regeneration.

### pPNP + TIIA@PFS-mediated promotion of chondrogenic differentiation in vivo

The pPNP + TIIA@PFS complex was meticulously engineered to optimize biocompatibility and facilitate cartilage integration. To preliminarily assess its safety profile, we conducted cytotoxicity assays and found no significant alterations in chondrocyte cell viability following treatment with varying concentrations of pPNP in vitro (Fig. S11). The variation in proportion of viable chondrocytes among these cultures on day 3 post-treatment underscores the robust cytocompatibility of pPNP + TIIA@PFS, even under extended culture periods (Fig. S12). Additionally, we conducted blood and histological analyses on treated rats to evaluate the potential systemic toxicity of degradation byproducts (Fig. S13). The results of these analyses in rats receiving pPNP + TIIA@PFS injections closely resembled those of healthy rats. These findings affirm the favorable biocompatibility of pPNP + TIIA@PFS.

**Fig. 7:**
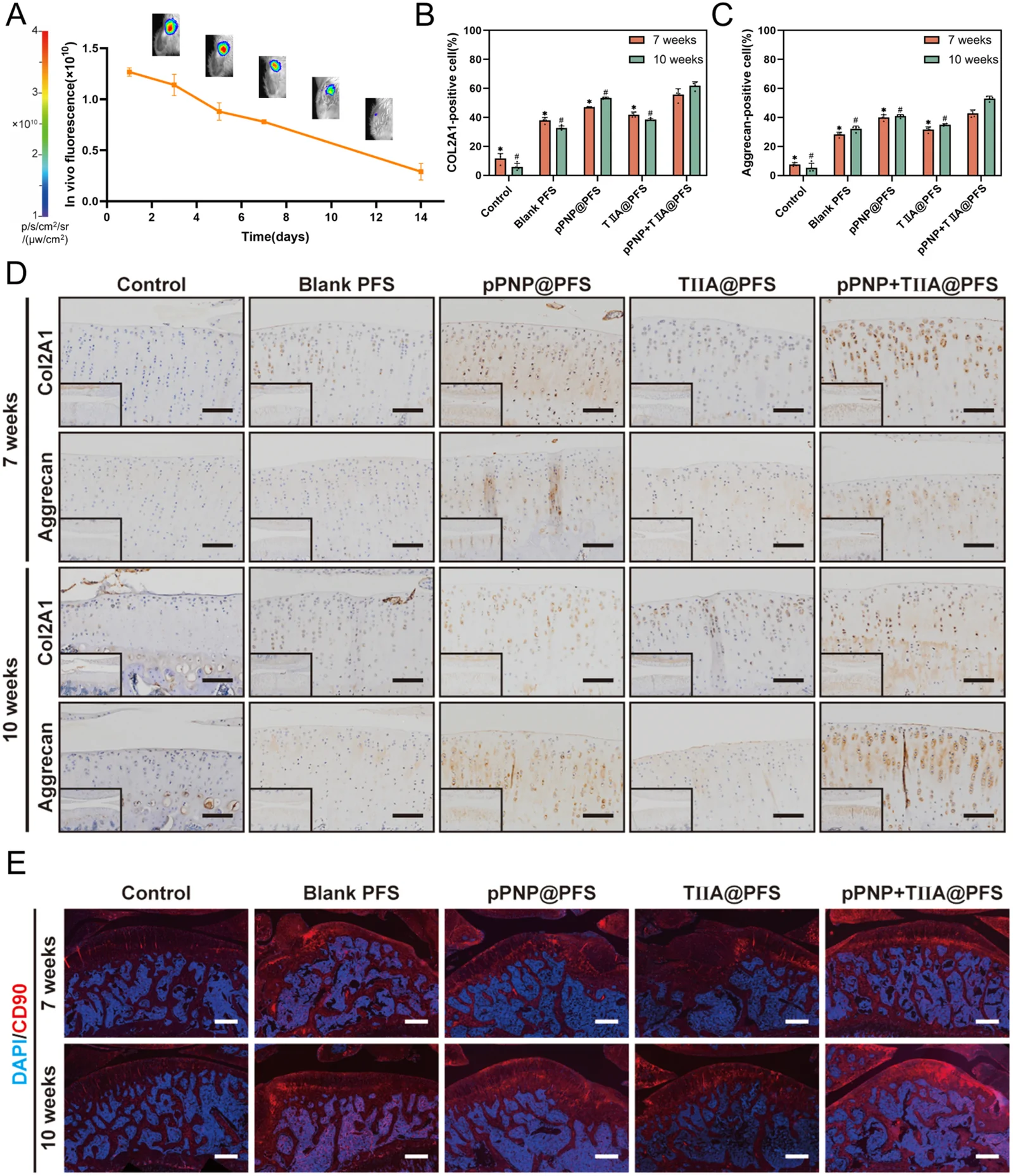
pPNP + TIIA@PFS-mediated promotion of chondrogenic differentiation in vivo. (**A**) In vivo biodegradation of pPNP + TIIA@PFS labeled with Cy5, visualized using in vivo fluorescence imaging of knee at day 14 post-injection (*n* = 4). (**B**, **C**) Quantification of cells with histological positivity for Col2A1 (**B**) and Aggrecan (**C**) (*n* = 4). (**D**) Representative images of immunohistochemistry staining of Col2A1 and Aggrecan in rat knee joints at 7 and 10 weeks post-ACLT surgery. Scale bar, 200 μm. (**E**) Immunofluorescence staining of CD90 in rat knee joints at 7 and 10 weeks. Nuclei were stained with DAPI. Scale bar, 500 μm. Data are presented as the mean ± SD. *P < 0.05 versus pPNP + TIIA@PFS group at 7 weeks, ^#^*P* < 0.05 versus pPNP + TIIA@PFS group at 10 weeks

We employed Cy5 labeling of the hydrogel to assess the biodegradability of pPNP + TIIA@PFS in the ACLT rat model. Although we observed a gradual attenuation of the fluorescence signal, it was nonetheless still discernable on day 14 post-injection (Fig. 7A). We also employed immunohistochemistry staining to investigate the expression of chondrogenic markers in the knee joints. Levels of chondrogenesis-related cytokines, specifically Col2A1 and Aggrecan, were significantly elevated following pPNP + TIIA@PFS treatment. Quantitative analyses further substantiated an increase in the numbers of Col2A1- and Aggrecan-positive cells within the pPNP + TIIA@PFS group compared to those in the other groups, indicating augmented synthesis of extracellular matrix (Fig. 7B–D). Subsequently, we conducted immunostaining of the joints for CD90. Indeed, the pPNP + TIIA@PFS group exhibited a clustered or sheet-like distribution of BMSCs around the articular surface (Fig. 7E). BMSCs were observed infiltrating the cartilage surface, suggesting potential differentiation into chondrocytes. Taken together, our findings strongly suggest that pPNP + TIIA@PFS substantially enhances cartilage regeneration.

## Discussion

Current disease-modifying drugs for OA often fall short in achieving desired therapeutic outcomes, primarily because they ameliorate symptoms rather than halt ongoing tissue deterioration [10]. Recent evidence underscores the role of cell senescence in OA development [41]. Senescent chondrocytes hinder the effectiveness of existing treatments to prevent cartilage degradation by increasing the activation of matrix-degrading enzymes and reducing the regenerative ability of chondrocytes. Consequently, selective targeting of senescent cells as a way to address the root cause of OA has gained attention. Several drugs and biologics have shown excellent results in modifying OA pathology in animal models [42, 43]. However, this approach poses challenges in drug delivery and carries the risk of excessively depleting scarce chondrocyte populations and accelerating stem cell exhaustion [44].

Recent advancements in hydrogel technology for OA therapy have shown promising results. Adhesive hydrogels leverage their strong adhesive properties to remain in place within the joint cavity, promoting repair and reducing inflammation [45, 46]. However, given the intricate nature of OA and its complex pathophysiology, incorporating biological functions into OA therapy is crucial for achieving optimal therapeutic outcomes. Self-assembled peptides (SAPs), which are conjugates of hydrophilic and hydrophobic groups, can self-assemble into aligned nanostructures [47]. SAPs have been widely used in regenerative medicine due to their ability to support cell attachment, migration, and differentiation, as well as to increase the residence time of implanted cells [48, 49]. The pPNP + TIIA@PFS complex, with its favorable lubricating properties, not only protects the joints but also enhances the retention of therapeutic agents, including small molecules and genetic material, within the joint cavity. This ensures targeted delivery at the cellular level and improved penetration into cartilage tissue. Additionally, the biodegradable nature of the self-assembled peptide hydrogel ensures minimal long-term accumulation and potential toxicity.

Recent advances in gene therapy have showcased the potential to attenuate chondrocyte senescence [50]. Our intraluminal injection of a NP-hydrogel hyper-structure allows for the introduction of *KL* into the nucleus of articular chondrocytes, which then produce the age-protective protein Klotho, thereby inhibiting senescence and improving the repair capacity of joints with OA [51, 52]. Klotho, a longevity-associated protein, is believed to play crucial roles in regulating the processes of aging, oxidative stress, and inflammation, particularly in the context of OA [53]. Furthermore, the gradual decline in Klotho protein levels with advancing age correlates with an exacerbation in the severity of OA [54]. This relationship underscores the potential significance of Klotho in mitigating age-related degenerative conditions. The delivery of *KL* synergistically combines regulation of senescence-related genes with augmentation of the chondrocyte population to effectively address the underlying factors contributing to cartilage degradation in OA.

The present study postulates that the synergistic actions of Klotho protein and TIIA exert a positive influence on cartilage regeneration in OA, as evidenced at both the cellular and genetic levels. Treatment with pPNP- and TIIA-embedded hydrogels has led to a significant attenuation in chondrocyte senescence, accompanied by a suppression of senescence signaling to adjacent healthy cells. Notably, the optimal improvement in articular cartilage morphology was achieved via injection of the pPNP + TIIA@PFS complex. This synergistic interaction manifests in two key dimensions. First, Klotho and TIIA produce complementary effects. Chondrocyte senescence and metabolic imbalances arise in response to a combination of cell cycle arrest and intensified growth signaling in damaged cartilage [55]. Klotho ameliorates related gene profile changes by downregulating the expression of p16^INK4a^, P21, and MMPs, thereby inhibiting matrix degradation and additional cartilage damage. Conversely, TIIA’s inhibition of CCN1 dampens the communication from aging chondrocytes to surrounding cells, weakening intercellular signaling and inhibiting age-induced morphological changes [56]. In the absence of TIIA-mediated regulation, growth signals emanating from damaged cartilage may induce senescence in adjacent healthy cells [57]. Similarly, in the absence of Klotho, merely addressing the metabolic imbalance would likely be insufficient to halt cartilage deterioration. Second, pPNP + TIIA treatment prompted significant alterations in gene expression pertaining to cellular senescence, adhesion, migration, and chondrogenesis, corroborating findings from both in vitro and in vivo studies. Prior research focusing on gene and stem cell therapies for OA has generally been limited to examining the individual impacts of these two approaches. In contrast, there is a dearth of studies exploring the combination of synergistic mechanisms in the development of therapeutic strategies for OA. We found that the combination of Klotho and TIIA significantly ameliorated the state of existing articular cartilage, decelerated age-related deterioration, and hindered the rapid progression of OA. Thus, our strategy offers a novel and effective therapeutic modality that capitalizes on the synergistic properties of Klotho and TIIA.

Achieving therapeutic efficacy with gene therapy requires the targeted delivery of an expression plasmid to the intended site of action. Unlike traditional adenoviral drug delivery methods, hydrogels provide biodegradable and injectable aqueous matrices [58, 59]. To achieve precise cartilage targeting and efficient nuclear-targeted gene delivery, we designed and synthesized pPNP. An amphiphilic peptide-SA monomer, (SA)_2_KRRRR-NLS-WYRGRL, was engineered by integrating cartilage-targeting sequences [60] and an NLS [61] as hydrophilic moieties, with SA as the hydrophobic structural domain, thereby overcoming the potential challenges of off-target cytotoxicity and drug accumulation. The hydrogel-based delivery system ensures sustained bioactivity and extended release of the *KL* plasmid (pKlotho), significantly improving its potential effectiveness in treating chronic diseases such as OA. Concurrently, NP structures that include an NLS offer a promising approach to the complex problem of DNA nucleation. Compared to intra-articular blank hydrogel injection, pPNP@PFS substantially improved cartilage integrity, cellular senescence, and joint viability over a 10-week period. The pPNPs remain stably bound within the PFS via electrostatic interactions with cations, ensuring consistent in vitro release over 40 days. Chondrocytes and BMSCs displayed favorable morphology and strong proliferation on PFS substrates, confirming the lack of cytotoxic effects. Although articulatory movements may degrade the implanted hydrogel, leading to faster release of pPNP in vivo, dynamic interactions between the PFS peptide and pPNP could potentially protect against pPNP displacement within the hydrogel matrix. Our injectable PFS-hydrogel delivery system not only enables sustained release of pKlotho, it also provides an advantageous in situ microenvironment. This microenvironment allows for controlled, minimally invasive gene vector delivery with both temporal and spatial precision. Importantly, it also restricts vector dispersion within the intra-articular space, thereby reducing the risk of loss of the therapeutic gene product [62].

An ideal hydrogel should precisely align with the cartilage regeneration process, ensuring effective chondrogenesis at the joint. Repairing articular cartilage damage is challenging due to the limited proliferative and regenerative capabilities of senescent chondrocytes, particularly in patients with OA. While exogenous mesenchymal stem cells (MSCs) serve as a feasible source of chondrogenic cells, a randomized controlled trial found no significant alterations in the whole-organ magnetic resonance imaging score of knee cartilage post-treatment, calling into question the efficacy of MSCs in cartilage repair [63]. Both culture-expanded MSCs and endogenous, joint-resident MSCs play crucial roles in repairing osteoarthritic joints. Functionalized hydrogels possess the ability to attract endogenous BMSCs and induce their differentiation into the chondrocytic lineage in vivo. To capitalize on this, we employed stem cell-homing peptides to recruit endogenous BMSCs and initiate cartilage regeneration. This strategy reduces dependence on exogenous cells and minimizes associated risks. Transwell assays conducted in vitro supported the role of PFS in promoting BMSC migration. Additionally, the stem cell-homing hydrogel facilitated BMSC differentiation into chondrocytes, as evidenced by the upregulated expression of Col2A1 and Aggrecan. One element of this strategy involves the extended release of Klotho and TIIA from PFS, a mechanism that maintains joint integrity by inhibiting chondrocyte senescence. Concurrently, the PFSSTKT peptide encapsulated within PFS enhances the neochondrocyte population by mobilizing endogenous BMSCs and facilitating chondrogenic differentiation [64, 65]. This integrated mechanism balances matrix catabolism and anabolism, presenting a judicious alternative to the prevention of inadvertent depletion of cellular populations in compromised cartilage offered by senescence-targeted interventions. Our approach was also found to support further rejuvenation of aged joints, leading to a post-treatment condition in OA-affected knee joints of ACLT model rats that closely resembled healthy joint physiology. Notably, the strategy yielded reductions in defect occurrence, preservation of cartilage integrity, and deceleration of degeneration, thus demonstrating its significant potential in the clinical management of OA.

Our study had limitations. First, we did not optimize the hydrogel formulation to explore the possibility that different hydrogel: pPNP ratios might yield improved therapeutic outcomes. Second, despite the powerful efficacy of the pPNP + TIIA@PFS system, it is imperative that comprehensive, long-term safety assessments are undertaken before contemplating clinical translation.

## Conclusions

We developed a pPNP + TIIA@PFS system that was found to modulate aging, prevent cartilage degeneration, and promote matrix regeneration, thereby serving as an effective therapeutic agent for OA. The combination of pPNP and TIIA in this system adjusted the dysregulated balance between extracellular matrix synthesis and the inflammatory phenotype in senescent chondrocytes and OA-affected joints. The hydrogel also attracted BMSCs and induced their differentiation into chondrocytes, thereby expediting the repair of cartilage defects and fostering new cartilage formation without the need for exogenous stem cells. OA-affected joints that underwent treatment with pPNP + TIIA@PFS exhibited an improvement in cartilage quality that was comparable to healthy cartilage. Overall, injectable cell-free approaches offer significant advantages in terms of ease of use and practicality for clinical application, and hold promise for advancing treatments that can benefit patients afflicted with OA.

## Electronic supplementary material

Below is the link to the electronic supplementary material.

Supplementary Material 1

## Data Availability

No datasets were generated or analysed during the current study.
