# Correction: Attenuation of osteoarthritis progression via locoregional delivery of Klotho-expressing plasmid DNA and Tanshinon IIA through a stem cell-homing hydrogel

**DOI:** 10.1186/s12951-026-05166-8

**Published:** 2026-10-30

**Authors:** Peng Wang, Zhibo Zhao, Ziyang Li, Xiao Li, Benzhao Huang, Xiaoqing Lu, Shimin Dai, Shishuo Li, Zhentao Man, Wei Li

**Affiliations:** 1 Department of Joint Surgery, Shandong Provincial Hospital, Shandong University, Jinan, 250021 Shandong P. R. China; 2 Department of Joint Surgery, Shandong Provincial Hospital Affiliated to Shandong First Medical University, Jinan, 250021 Shandong P. R. China; 3 Department of Orthopedic Surgery, Second Affiliated Hospital, Zhejiang University School of Medicine, Hangzhou, China; 4 College of Sports Medicine and Rehabilitation, Shandong First Medical University & Shandong Academy of Medical Sciences, Jinan, 250021 Shandong P. R. China; 5 Endocrine and Metabolic Diseases Hospital of Shandong First Medical University, Shandong Institute of Endocrine and Metabolic Diseases, Jinan, 250062 Shandong P. R. China

**Correction: Journal of Nanobiotechnology (2024) 22:325**

**https://doi.org/10.1186/s12951-024-02608-z**

In this article Fig. 5 appeared incorrectly: A representative image from Group 2 (KLDL) was mistakenly placed in the position of Group 3 (PFS), causing the two groups to be confused in the figure.

This correction concerns only the representative image and does not affect the underlying experimental data, data analysis, reported results, discussion, or conclusions of the article.

For completeness and transparency, the old incorrect and correct versions of Fig. 5 are displayed below.

Incorrect Fig. 5.

**Fig. 5:**
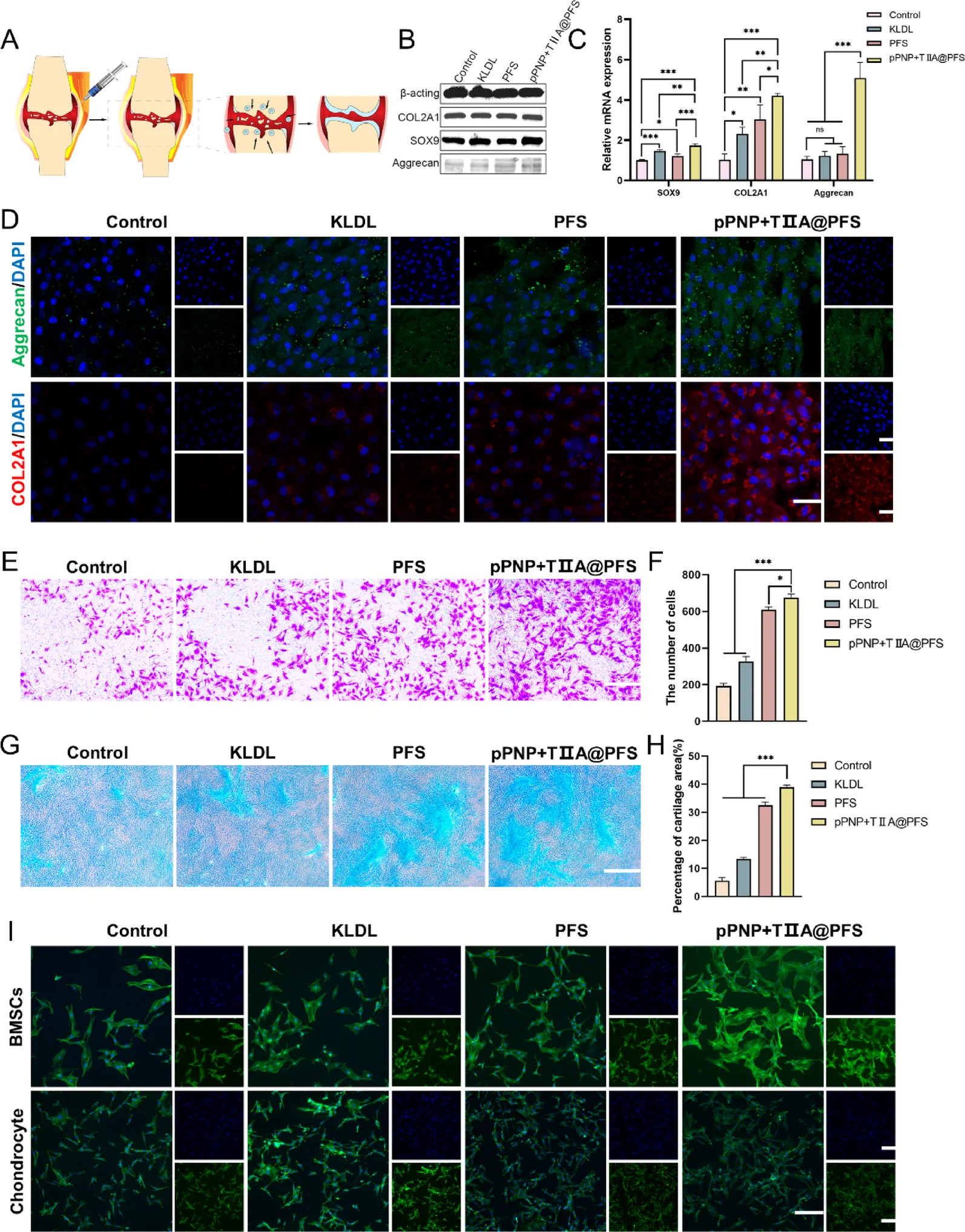
PFS@pPNP-mediated induction of BMSC recruitment and promotion of chondrogenic differentiation. (**A**) Schematic illustration of the recruitment process pattern of BMSCs during cartilage repair. (**B**, **C**) WB and qRT-PCR analyses of protein (**B**) and mRNA (**C**) levels of Sox9, Aggrecan, and Col2A1 in BMSC pellets in the indicated treatment groups (*n* = 3). (**D**) Immunofluorescence staining of Aggrecan and Col2A1 in BMSCs cultured on KLDL or PFS for 7 days. Scale bar, 40 μm. (**E**) Representative images of the Transwell bottom membrane stained with crystal violet after 24-h culture in the indicated treatment groups. Scale bar, 500 μm. (**F**) Quantification of cells observed at the bottom of the membrane (*n* = 3). (**G**) Alcian blue staining in BMSC pellets after 7-day culture in the indicated treatment groups. Scale bar, 250 μm. (**H**) Quantification analysis of chondrocyte area following Alcian blue staining (*n* = 3). (**I**) Alexa Fluor 488 phalloidin and DAPI staining showing morphology of BMSCs and chondrocytes grown under KLDL or PFS. Scale bar, 200 μm. Data are presented as the mean ± SD. *P < 0.05, **P < 0.01, ***P < 0.001. NS, not significant

Correct Fig. 5.

**Fig. 5:**
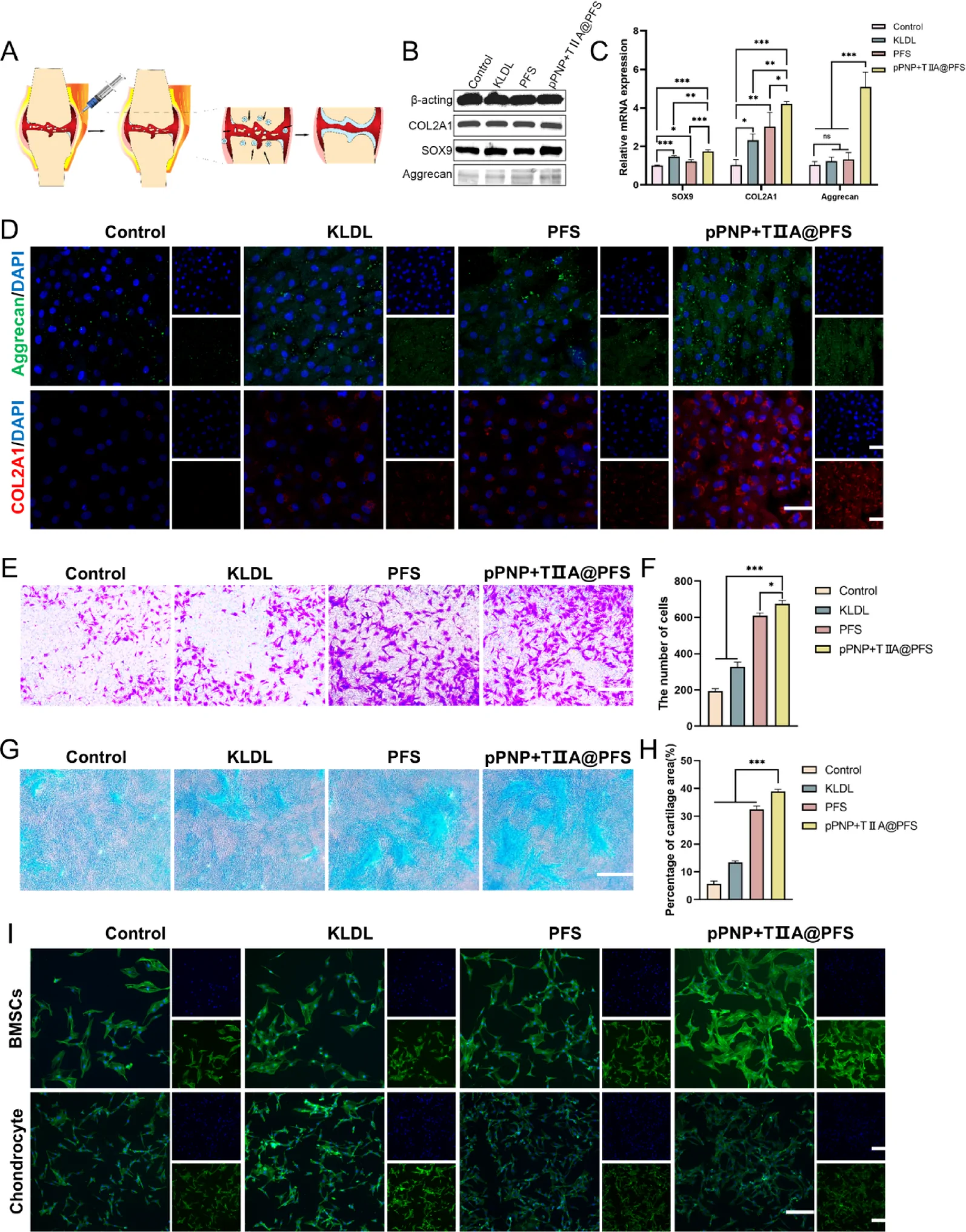
PFS@pPNP-mediated induction of BMSC recruitment and promotion of chondrogenic differentiation. (**A**) Schematic illustration of the recruitment process pattern of BMSCs during cartilage repair. (**B**, **C**) WB and qRT-PCR analyses of protein (**B**) and mRNA (**C**) levels of Sox9, Aggrecan, and Col2A1 in BMSC pellets in the indicated treatment groups (*n* = 3). (**D**) Immunofluorescence staining of Aggrecan and Col2A1 in BMSCs cultured on KLDL or PFS for 7 days. Scale bar, 40 μm. (**E**) Representative images of the Transwell bottom membrane stained with crystal violet after 24-h culture in the indicated treatment groups. Scale bar, 500 μm. (**F**) Quantification of cells observed at the bottom of the membrane (*n* = 3). (**G**) Alcian blue staining in BMSC pellets after 7-day culture in the indicated treatment groups. Scale bar, 250 μm. (**H**) Quantification analysis of chondrocyte area following Alcian blue staining (*n* = 3). (**I**) Alexa Fluor 488 phalloidin and DAPI staining showing morphology of BMSCs and chondrocytes grown under KLDL or PFS. Scale bar, 200 μm. Data are presented as the mean ± SD. *P < 0.05, **P < 0.01, ***P < 0.001. NS, not significant

The original article has been corrected.

