# Supplementary material for: Attenuation of osteoarthritis progression via locoregional delivery of Klotho-expressing plasmid DNA and Tanshinon IIA through a stem cell-homing hydrogel
Source: J Nanobiotechnology. 2024 Jun 10;22:325. doi: 10.1186/s12951-024-02608-z (PMC11163801; doi:10.1186/s12951-024-02608-z)
Supplement: Supplementary file 1 — Supplementary Material 1 [file 12951_2024_2608_MOESM1_ESM.docx]

**Attenuation of osteoarthritis progression via locoregional delivery of Klotho-expressing plasmid DNA and Tanshinon IIA through a stem cell-homing hydrogel**

Peng Wang^1 †^, Zhibo Zhao^1 †^, Xiao Li^1^, Ziyang Li^5^, Benzhao Huang^2^ ,Xiaoqing Lu^2^, Shimin Dai^2^, Shishuo Li^2^, Zhentao Man^1,2,3,4*^ and Wei Li^1,2,3*^

^†^Peng Wang and Zhibo Zhao are contributed equally to this work

^*^ Correspondence: Zhentao Man mztqd1984@163.com, Wei Li greatli2000@163.com

^1^Department of Joint Surgery, Shandong Provincial Hospital, Shandong University, Jinan, Shandong 250021, P. R. China

^2^Department of Joint Surgery, Shandong Provincial Hospital Affiliated to Shandong First Medical University, Jinan, Shandong 250021, P. R. China

^3^College of Sports Medicine and Rehabilitation, Shandong First Medical University & Shandong Academy of Medical Sciences, Jinan, Shandong 250021, P. R. China

^4^Endocrine and Metabolic Diseases Hospital of Shandong First Medical University, Shandong Institute of Endocrine and Metabolic Diseases, Jinan, Shandong 250062, P. R. China

^5^Department of Sports Medicine, Zhejiang University School of Medicine, Hangzhou, Zhejiang 310058, P. R. China


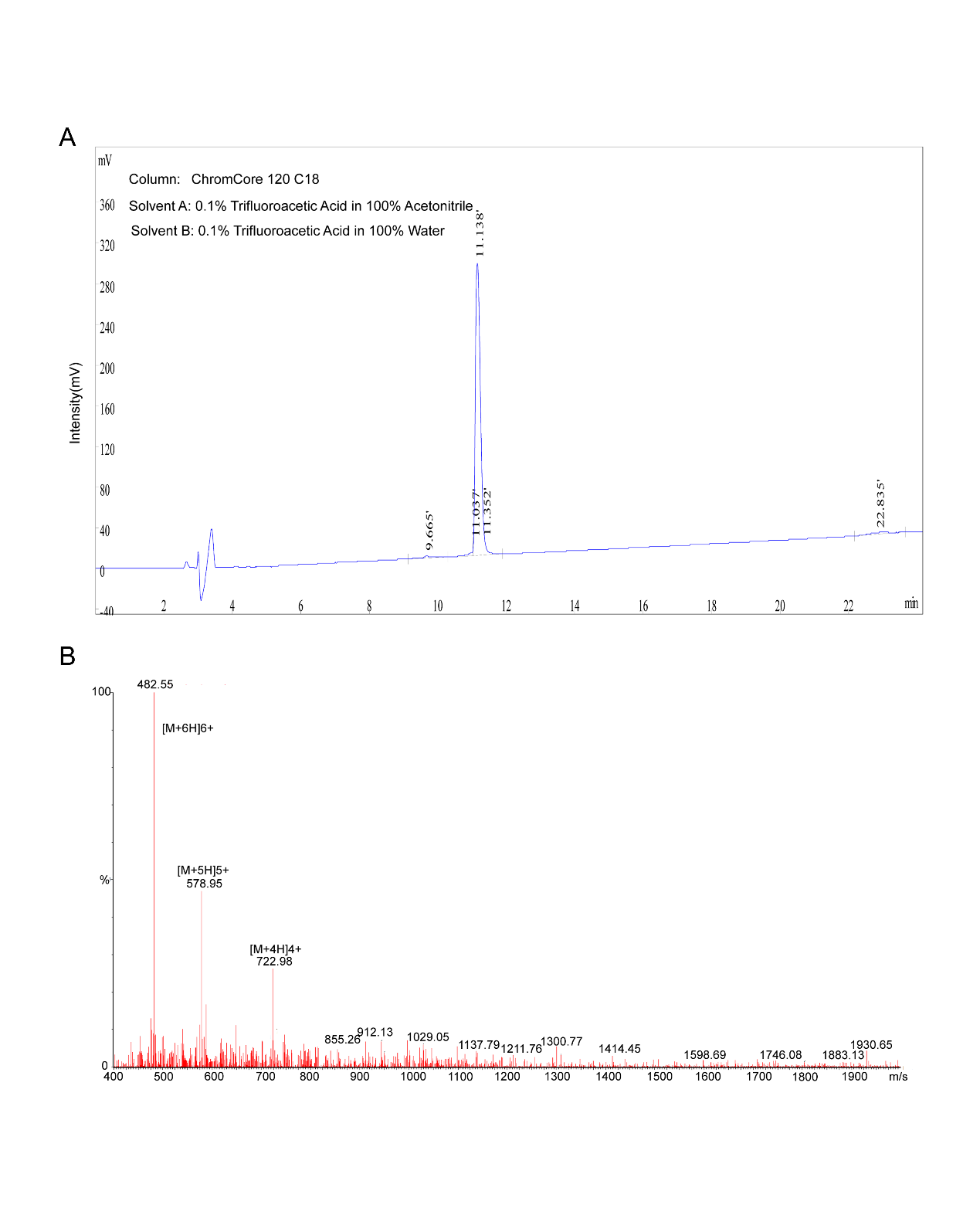


**Figure S1. Characterization of the amphiphilic peptide-SA monomer.** (A) The purity of the peptide as confirmed by HPLC. (B) The molecular weight of the peptide-SA monomer as certified by ESI-MS.


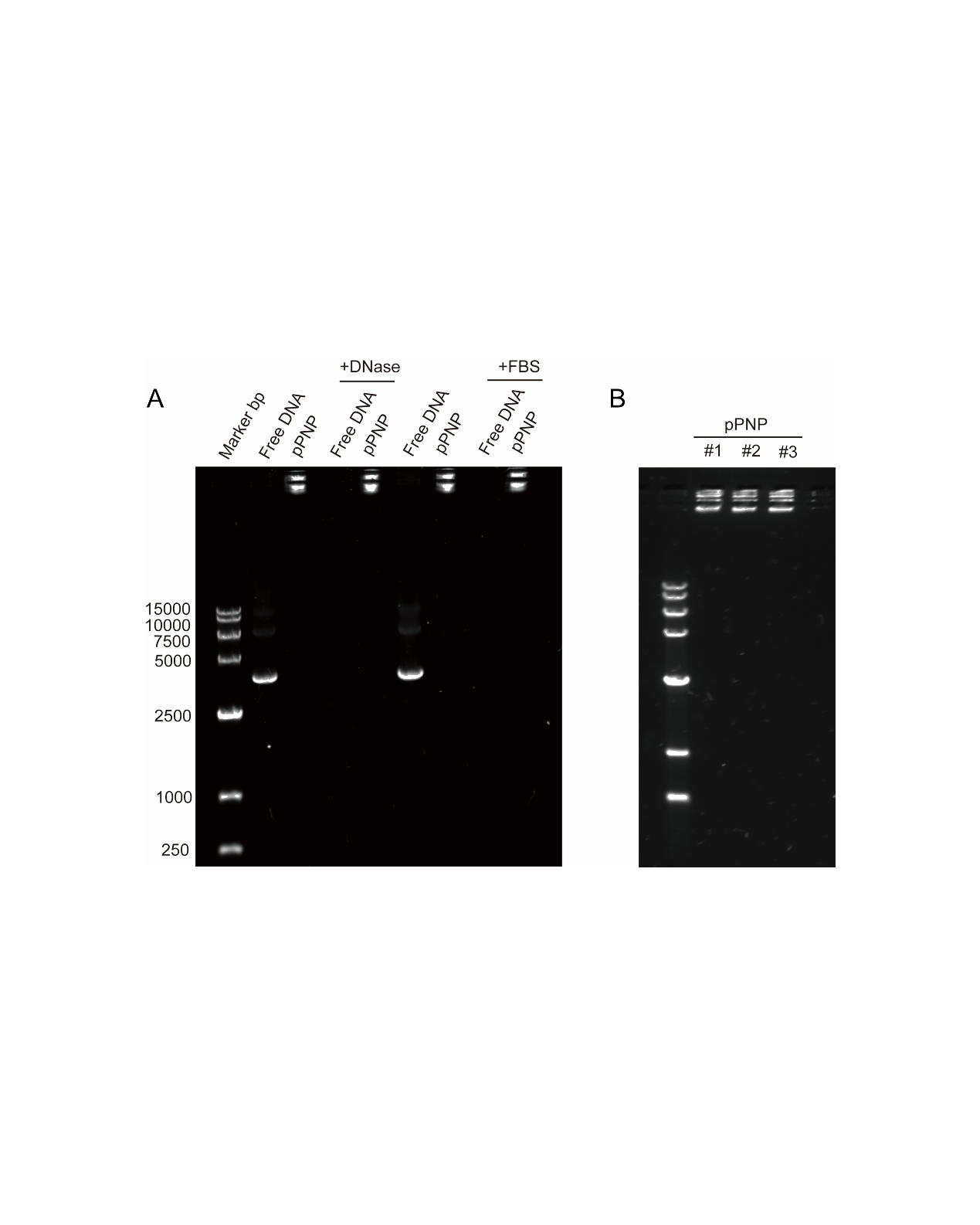


**Figure S2. Gel retardation assay of pPNP stability.** (A) The stability of pPNP at a weight ratio of peptide-SA monomers to pDNA of 10:1. DNase I digestion and FBS treatment were performed at 37°C for 1 h.(B) Gel retardation assay showing the stability of pPNP after 14 days. The experiments were repeated three times independently.


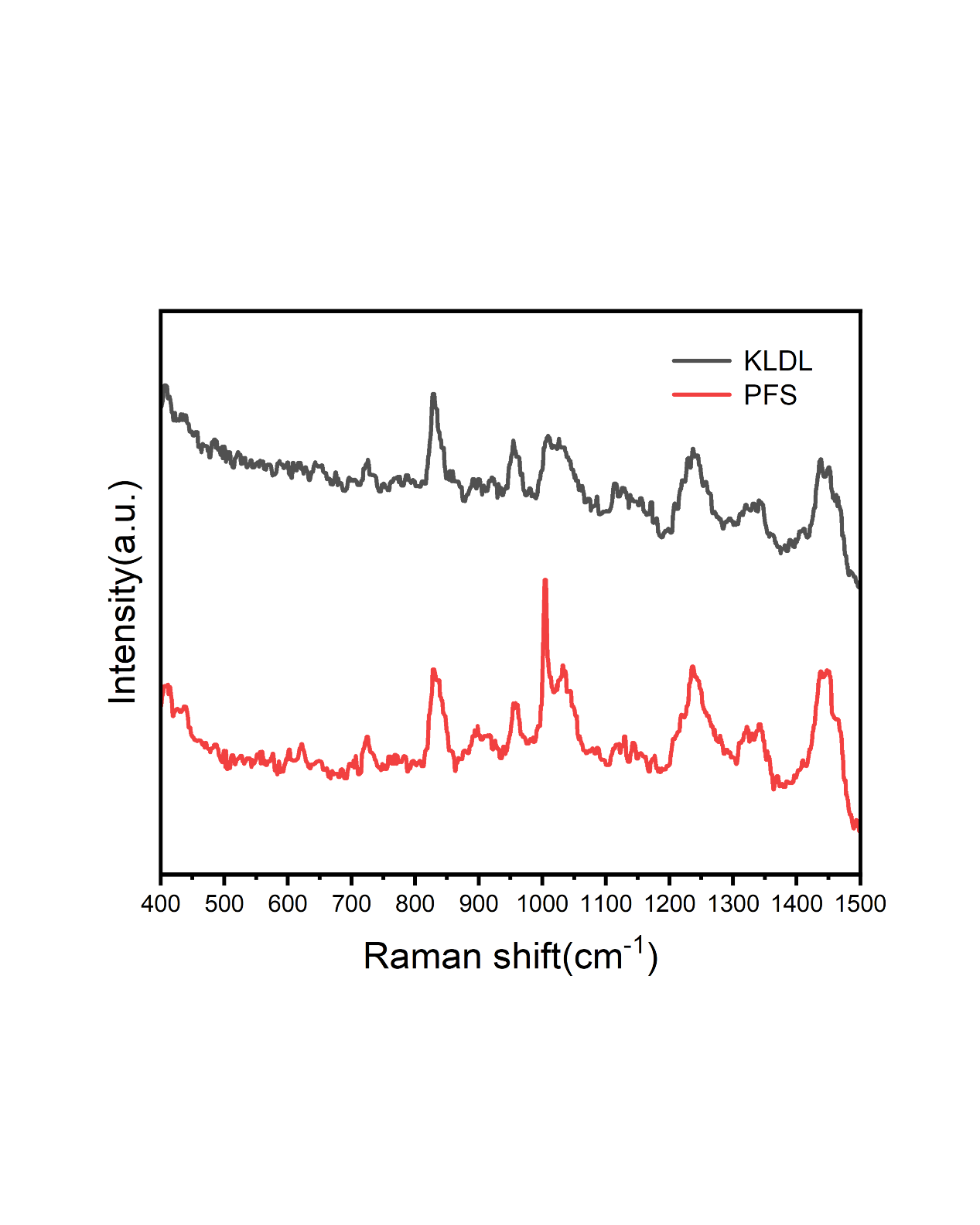


**Figure S3.** Raman spectra of freeze-dried KLDL/PFS hydrogel.


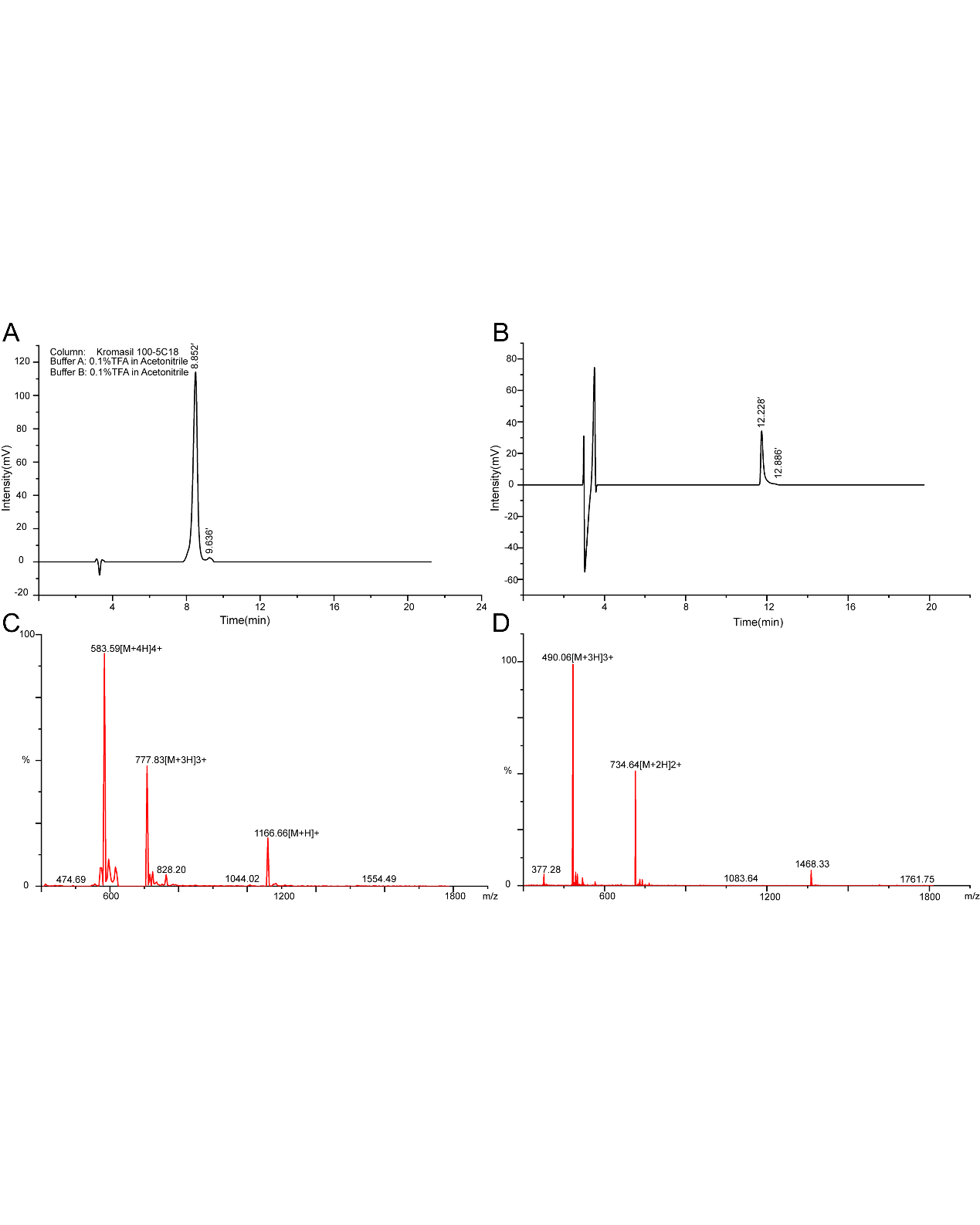


**Figure S4. Characterization of the self-assembling peptide hydrogel.** (A, B) The purity of KLDL-PFS (A) and KLDL (B) as confirmed by HPLC. (C, D) The molecular weight of KLDL-PFS (C) and KLDL (D) as certified by ESI-MS.


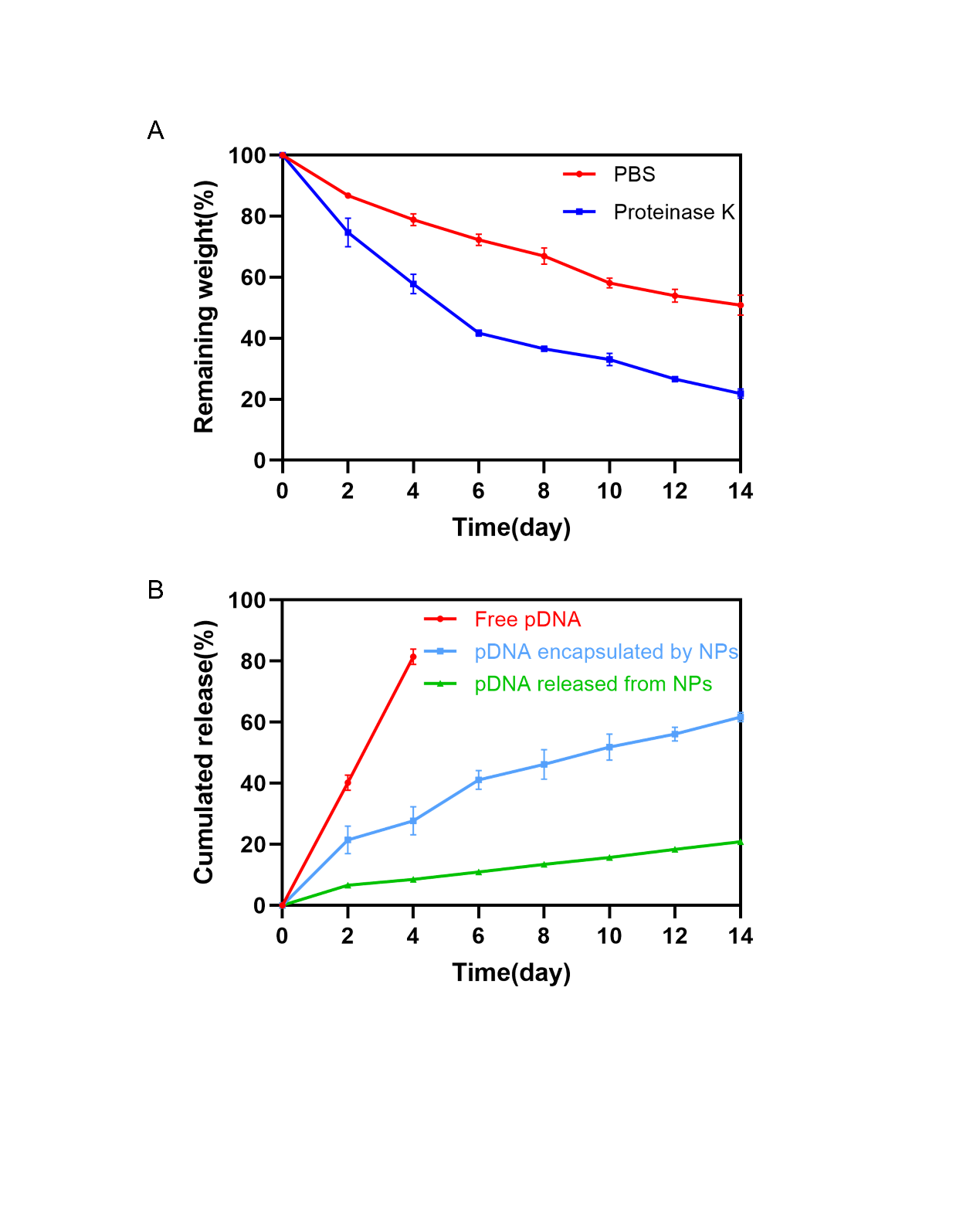


**Figure S5. Stability and release profile of NP-pKlotho in vitro.** (A) Degradation of the blank hydrogel in phosphate-buffered saline, with or without proteinase K digestion. (B) The release profile of pKlotho from the hydrogel. Data expressed as means ± SD (n = 3).

**
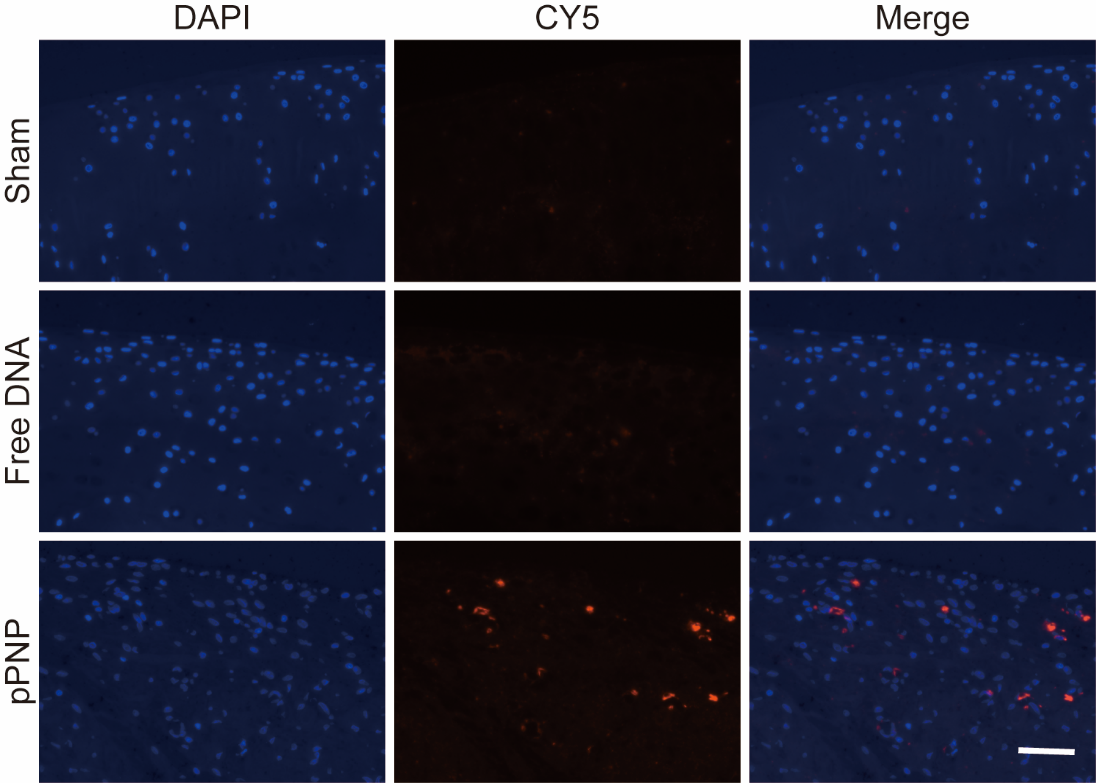
**

**Figure S6.** Immunofluorescence staining showing that pPNPs (red) in rat knee

cartilage after surgery. Scale bar, 75μm.

**
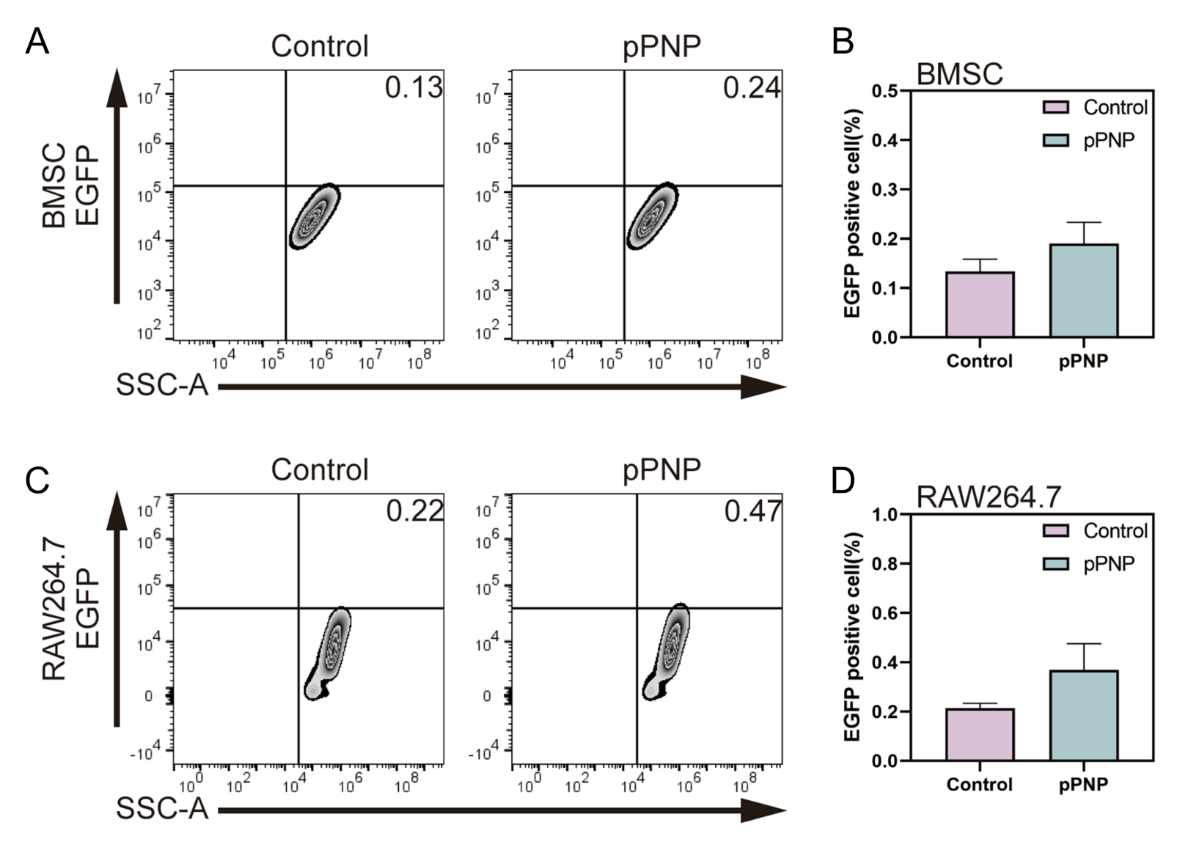
**

**Figure S7. Col2 promoter-driven, chondrocyte-specific *KL* transgene expression.** Transduction of rat BMSCs and RAW264.7 cells with pDNA including the Col2 promoter. (A, C) Percentage of EGFP-positive BMSCs (A) and RAW264.7 cells (C) after treatment with free pDNA or pPNPs. (B, D) Quantitative analysis of the flow cytometry data. Data expressed as means ± SD (n = 3 independent experiments per group).


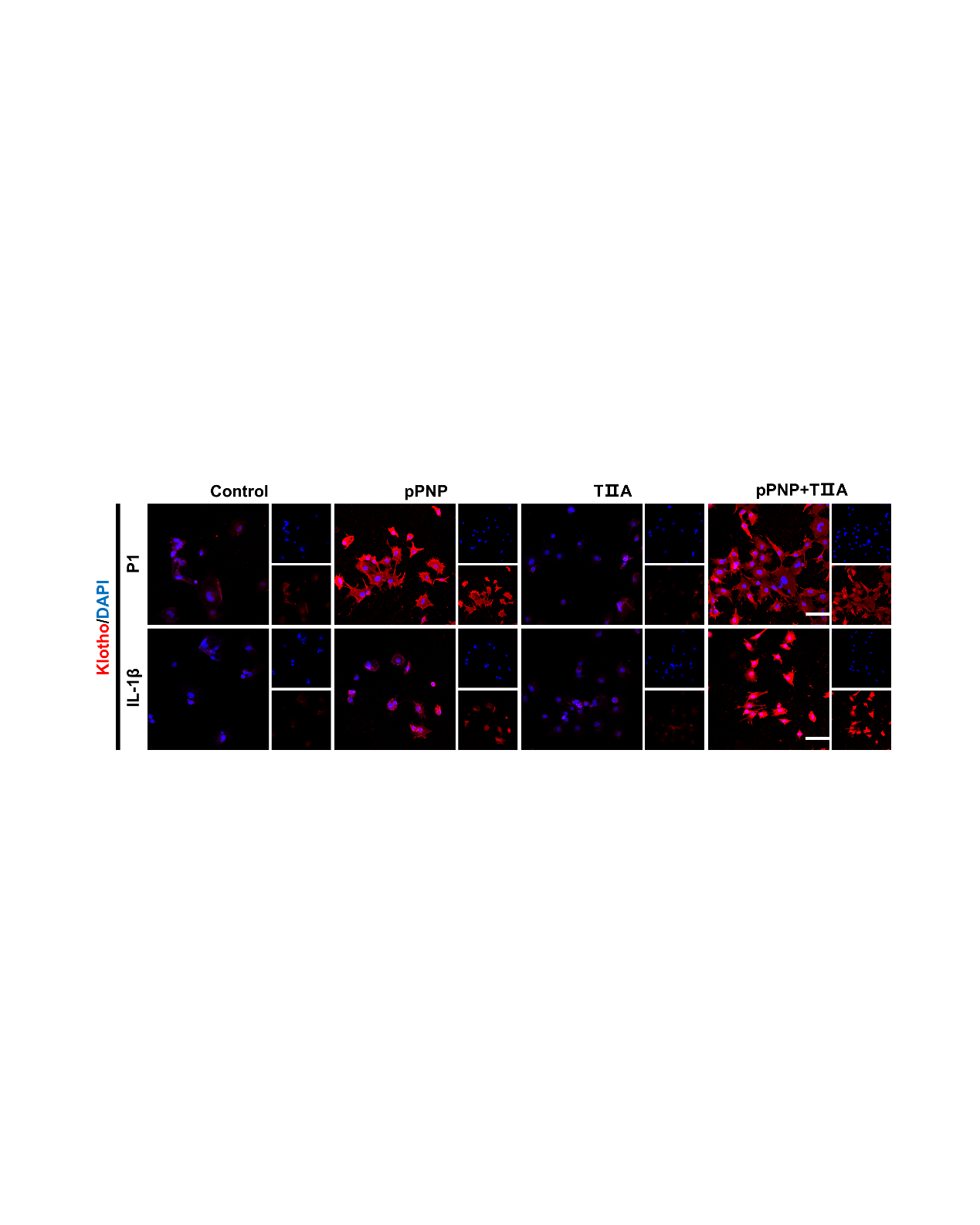


**Figure S8.** Immunofluorescence staining of Klotho in interleukin (IL)-1β-treated chondrocytes and chondrocytes at passage 1 (P1). Scale bar, 200 µm.

**
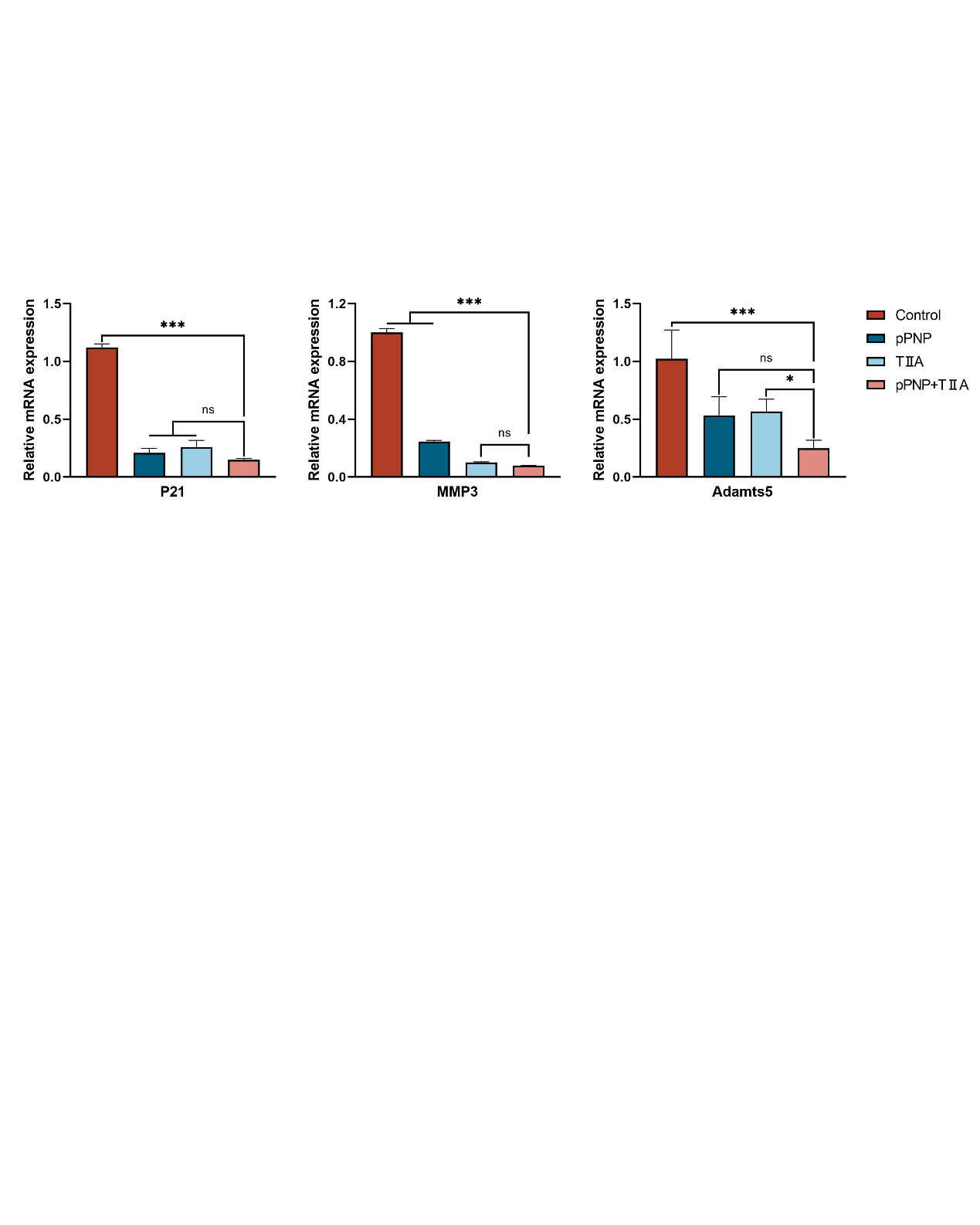
**

**Figure S9.** qPCR evaluation of the expression of P21, MMP3, and Adamts5 at 2 days post-transfection of rat chondrocytes with different cultures (n = 3).


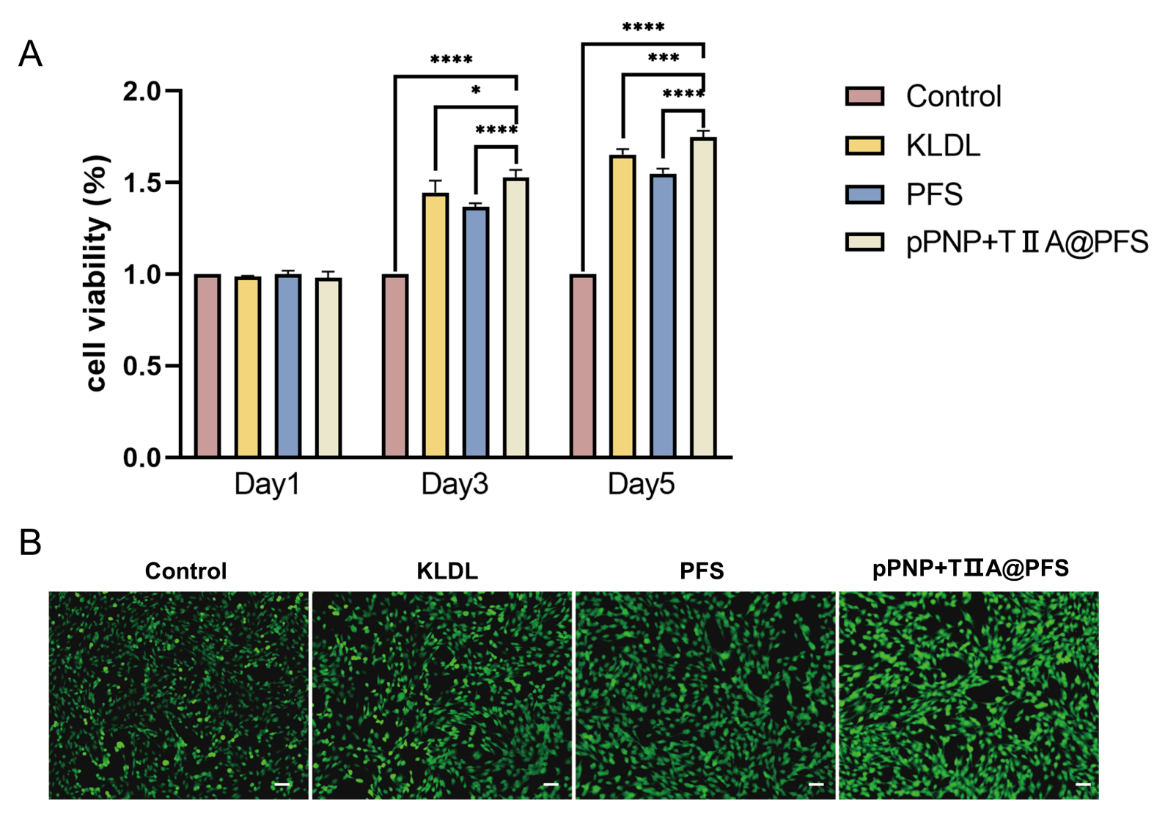


**Figure S10.** (A) CCK-8 viability assay of BMSC cultured under normal conditions, or in the presence of KLDL, PFS, or pPNP+TIIA@PFS (n = 3). (B) Representative fluorescence images of the proliferation of BMSC cultured for 3 days on different culture. Scale bar, 50 µm.

**
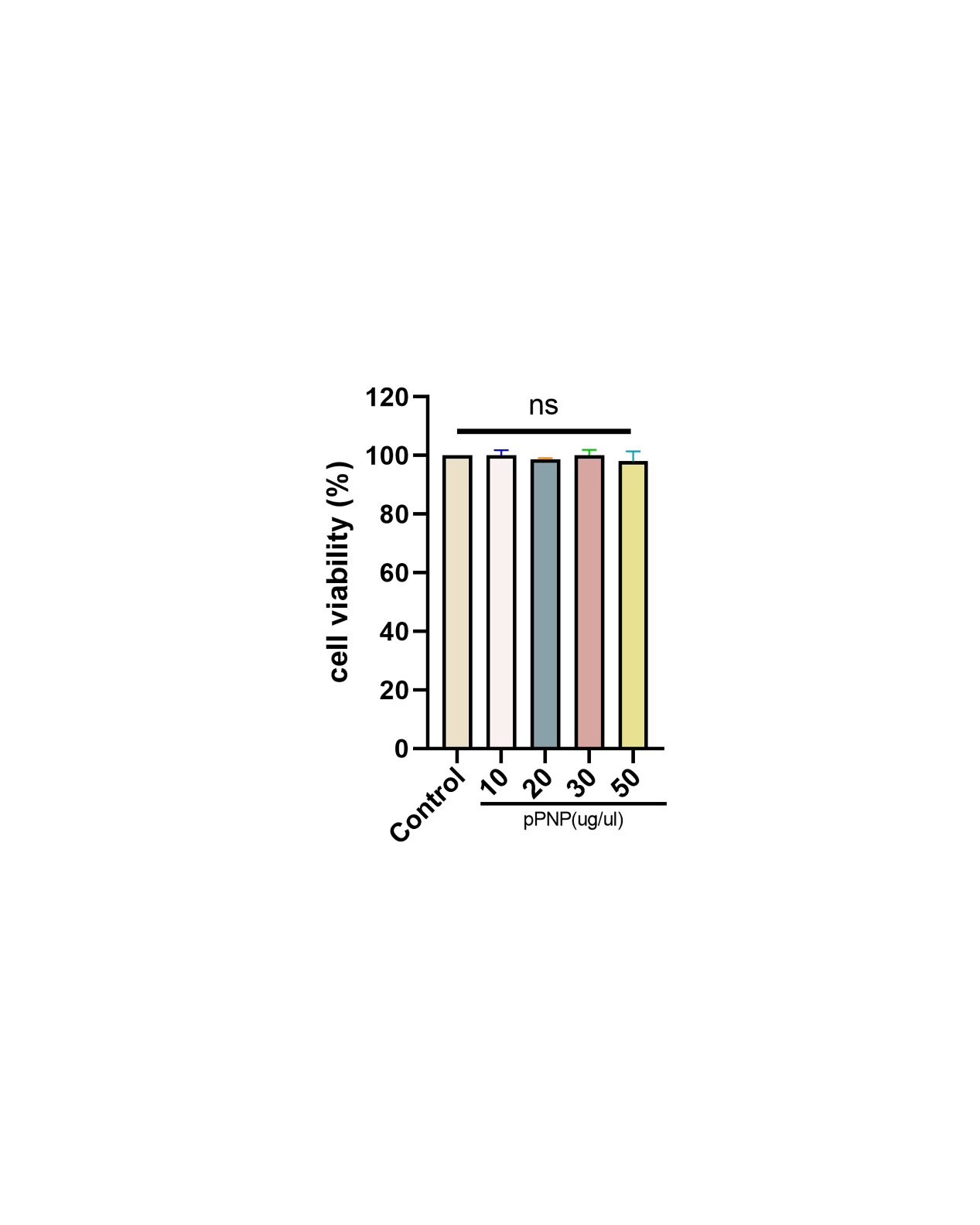
**

**Figure S11.** Cytotoxicity assays of pPNPs in chondrocytes (n = 3). NS, not significant.

**
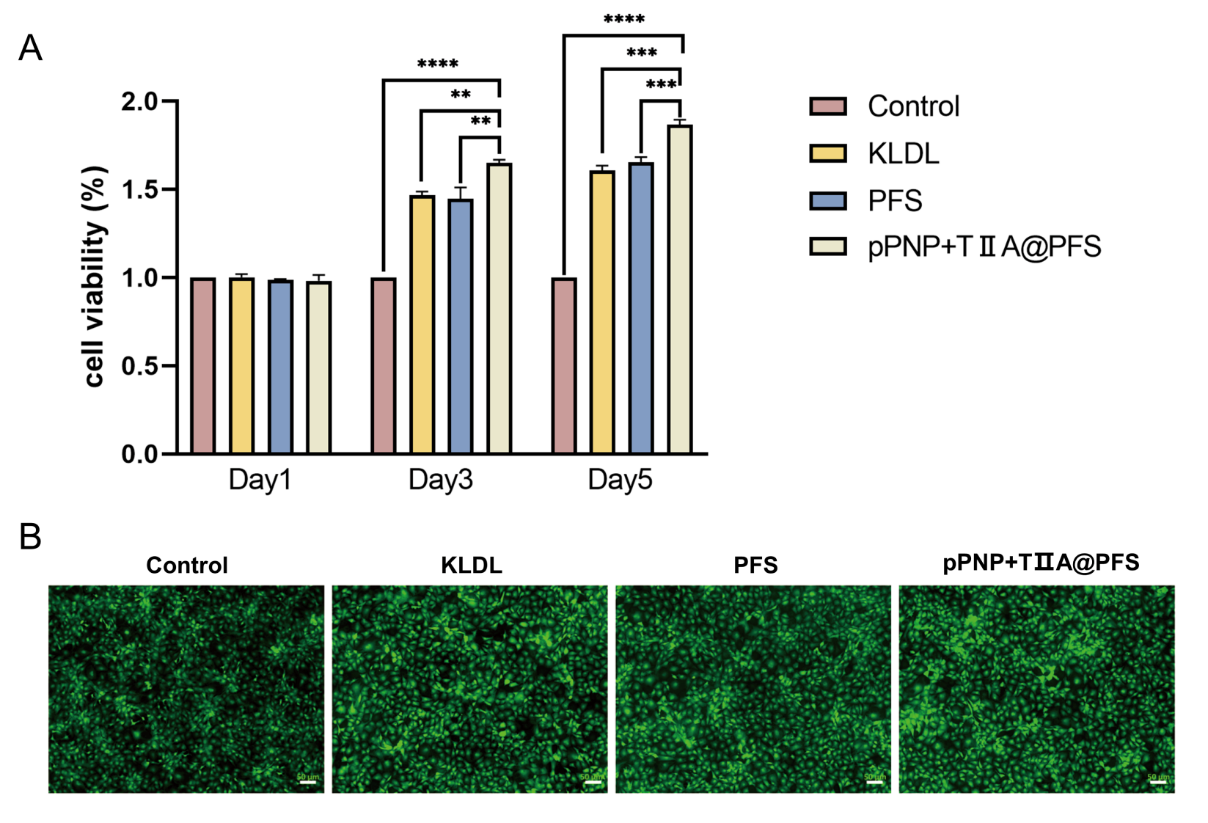
**

**Figure S12.** (A) CCK-8 viability assay of chondrocytes cultured under normal conditions, or in the presence of KLDL, PFS, or pPNP+TIIA@PFS (n = 3). (B) Representative fluorescence images of the proliferation of chondrocytes cultured for 3 days on different culture. Scale bar, 50 µm.

**
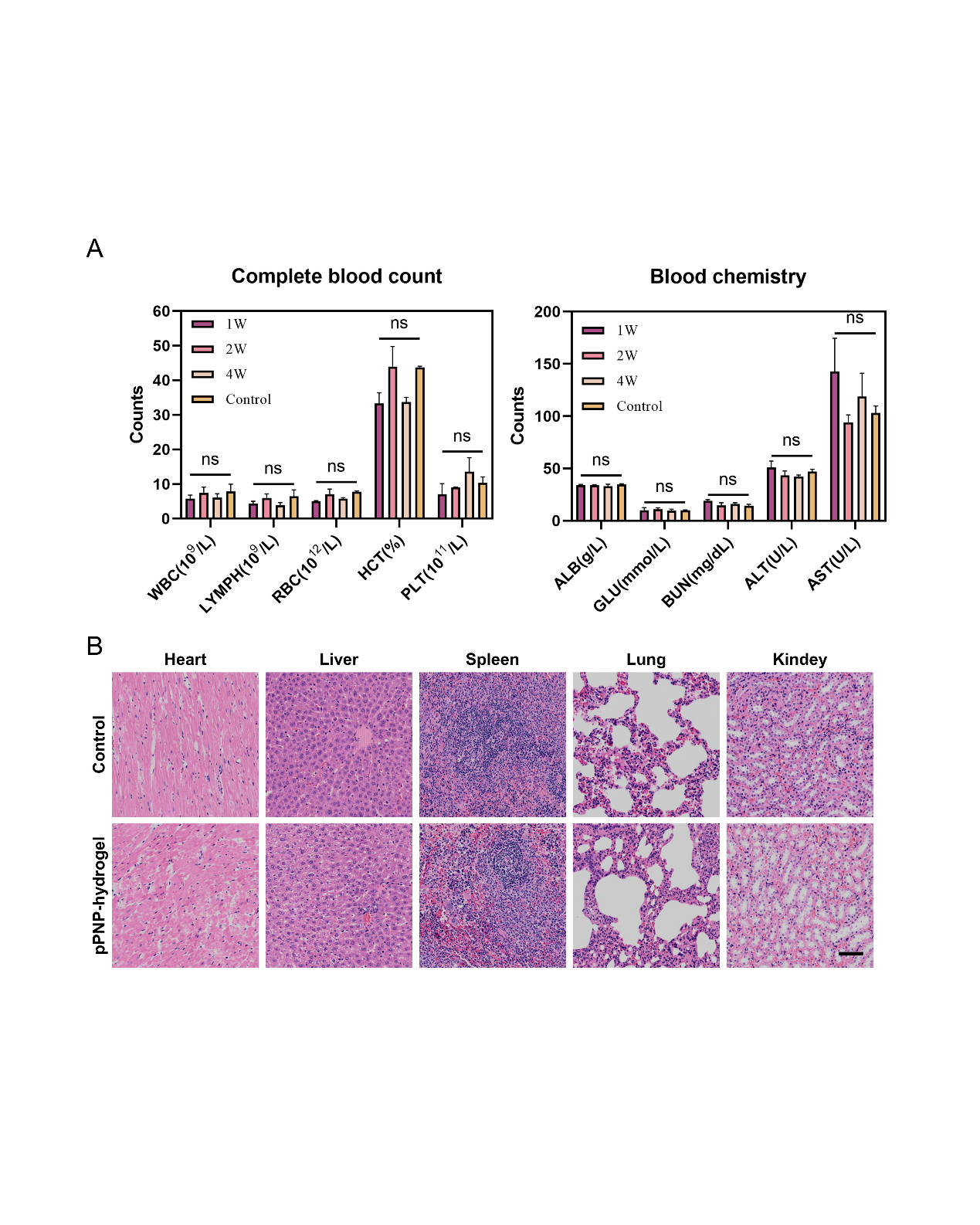
**

**Figure S13. Systemic toxicity evaluation of the** **pPNP+TIIA@PFS.** (A) Complete blood counts and blood chemistry analyses of healthy control rats (without surgery) and pPNP-hydrogel-injected rats. WBC, white blood cell; LYMPH, lymphocyte; RBC, red blood cell; HCT, hematocrit; PLT, platelet; ALB, albumin; GLU, glucose; BUN, blood urea nitrogen; ALT, alanine transaminase; AST, aspartate transaminase (n = 3). (B) Histological analysis of the major organs from healthy control rats (without surgery) and pPNP-hydrogel-injected rats. Scale bar, 500 µm. NS, not significant.

**Table S1.** Osteoarthritis Research Society International (OARSI) scores

| Grade (key feature) | Subgrade (optional) | Associated criteria (tissue reaction) |
| --- | --- | --- |
| Grade 0: surface intact,  cartilage intact | No subgrade | Intact, uninvolved cartilage |
| Grade 1: surface intact | 1.0 Cells intact | Matrix: superficial zone intact, edema and/or fibrillation |
|  | 1.5 Cells death | Cells: proliferation (clusters), hypertrophy  Reaction must be more than superficial fibrillation only |
| Grade 2: surface  discontinuity | 2.0 Fibrillation through superficial zone | As above |
|  | 2.5 Surface abrasion with matrix loss  within superficial zone | +Discontinuity at superficial zone  ±Cationic stain matrix depletion (Safranin  O or Toluidine Blue) upper 1/3 of cartilage  (mid zone)  ±Disorientation of chondron columns |
| Grade 3: vertical  fissures | 3.0 Simple fissures | As above |
|  | 3.5 Branched/complex fissures | ±Cationic stain depletion (Safranin O or  Toluidine Blue) into lower 2/3 of cartilage  (deep zone)  ±New collagen formation (polarized light  microscopy, Picro Sirius Red stain) |
| Grade 4: erosion | 4.0 Superficial zone delamination | Cartilage matrix loss, cyst formation within  cartilage matrix |
|  | 4.5 Mid zone excavation |  |
| Grade 5: denudation | 5.0 Bone surface intact | Surface is sclerotic bone or reparative tissue  including fibrocartilage |
|  | 5.5 Reparative tissue surface present |  |
| Grade 6: deformation | 6.0 Joint margin osteophytes | Bone remodelling. Deformation of articular  surface contour (more than osteophyte  formation only) |
|  | 6.5 Joint margin and central osteophytes | Includes: microfracture and repair |

**Table S2.** Related information about primary antibodies

| **Product name** | **Species Reactivity** | **Molecular weight** | **Source** |
| --- | --- | --- | --- |
| **GAPDH** | Rabbit | 37 kDa | CST, America |
| **β-actin** | Rabbit | 45 kDa | CST, America |
| **Mmp13** | Rabbit | 54 kDa | Abcam, England |
| **Sox 9** | Rabbit | 56 kDa | Abcam, England |
| **Col2A1** | Rabbit | 142 kDa | Abcam, England |
| **Aggrecan** | Rabbit | 250 kDa | Proteintech, China |
| **Klotho** | Mouse | 116 kDa | Proteintech, China |
| **P21** | Rabbit | 21 kDa | CST, America |
| **P16^ink4a^** | Rabbit | 16 kDa | CST, America |
| **Adamts5** | Rabbit | 73 kDa | ABclonal, China |
| **CCN1** | Rabbit | 41 kDa | Abcam, England |

**Table S3.** Sequences of the primers used in this study

| **Gene** | **Forward primer (5′-3′)** | **Reverse primer (5′-3′)** |
| --- | --- | --- |
| **Mmp13** | CATCATCTGGGAGCATGAAA | GCAGCTCCAAAGGCTACAA |
| **Mmp3** | AGTGCTTCTGAATGTCCTTCG | TCTTCCTCTGAAACTTGGCG |
| **Adamts5** | TCGTGGCCGCGTTCTTGCTCAC | ACGCGGGACCTCAGACGTGGTG |
| **P16^ink4a^** | GCGTTGCCAGAAGTGAAGCCA | CGTCGTGCGGTATTTGCGGTAT |
| **P21** | GCTCTGGACGGTACGCTTAGGT | CTGCCTGGTTCCTTGCCACTTC |
| **Sox9** | GCACCAGGGTCCAGTCATA | TAAATTCCCAGTGTGCATCC |
| **Col2A1** | CCTGGACCCCGTGGCAGAGA | GCAGGGCCAGAAGTACCCTGATC |
| **Aggrecan** | TGGGGTCCGTGGGCTCACAA | CATTCGCACGGGAGCAGCCA |
| **Klotho** | CTCTGAAAGCCTACGTGTTGG | TAGAAACGAGATGAAGGCCAG |
| **β-actin** | TGTCACCAACTGGGACGATA | GGGGTGTTGAAGGTCTCAAA |
